# N-Termini of Fungal CSL Transcription Factors Are Disordered, Enriched in Regulatory Motifs and Inhibit DNA Binding in Fission Yeast

**DOI:** 10.1371/journal.pone.0023650

**Published:** 2011-08-12

**Authors:** Martin Převorovský, Sophie R. Atkinson, Martina Ptáčková, Janel R. McLean, Kathleen Gould, Petr Folk, František Půta, Jürg Bähler

**Affiliations:** 1 Department of Genetics, Evolution & Environment and UCL Cancer Institute, University College London, London, United Kingdom; 2 Department of Cell Biology, Faculty of Science, Charles University in Prague, Prague, Czech Republic; 3 Howard Hughes Medical Institute and Department of Cell and Developmental Biology, Vanderbilt University School of Medicine, Nashville, Tennessee, United States of America; University of Kent, United Kingdom

## Abstract

**Background:**

CSL (CBF1/RBP-Jκ/Suppressor of Hairless/LAG-1) transcription factors are the effector components of the Notch receptor signalling pathway, which is critical for metazoan development. The metazoan CSL proteins (class M) can also function in a Notch-independent manner. Recently, two novel classes of CSL proteins, designated F1 and F2, have been identified in fungi. The role of the fungal CSL proteins is unclear, because the Notch pathway is not present in fungi. In fission yeast, the Cbf11 and Cbf12 CSL paralogs play antagonistic roles in cell adhesion and the coordination of cell and nuclear division. Unusually long N-terminal extensions are typical for fungal and invertebrate CSL family members. In this study, we investigate the functional significance of these extended N-termini of CSL proteins.

**Methodology/Principal Findings:**

We identify 15 novel CSL family members from 7 fungal species and conduct bioinformatic analyses of a combined dataset containing 34 fungal and 11 metazoan CSL protein sequences. We show that the long, non-conserved N-terminal tails of fungal CSL proteins are likely disordered and enriched in phosphorylation sites and PEST motifs. In a case study of Cbf12 (class F2), we provide experimental evidence that the protein is proteolytically processed and that the N-terminus inhibits the Cbf12-dependent DNA binding activity in an electrophoretic mobility shift assay.

**Conclusions/Significance:**

This study provides insight into the characteristics of the long N-terminal tails of fungal CSL proteins that may be crucial for controlling DNA-binding and CSL function. We propose that the regulation of DNA binding by Cbf12 via its N-terminal region represents an important means by which fission yeast strikes a balance between the class F1 and class F2 paralog activities. This mode of regulation might be shared with other CSL-positive fungi, some of which are relevant to human disease and biotechnology.

## Introduction

Transcription factors are important and well-studied regulators of gene expression. Accordingly, cells need to tightly control transcription factors at multiple levels, and by multiple mechanisms, to correctly coordinate biological processes. Such control is achieved by the orchestrated action of, for example, protein phosphorylation, proteolytic processing, protein-protein interactions or subcellular localization (e.g., [1], [2]).

Intrinsic protein disorder reflects the lack of a well-defined 3-dimensional structure *in vivo*, which is highly prevalent across phyla, and its degree correlates with organism complexity [3]. Disordered regions are typically highly accessible and can serve as sites of post-translational modifications, proteolysis, or docking sites for other proteins. They have also been associated with regulatory processes such as transcription, cell cycle control or differentiation, and with disease states [4]–[6].

CSL (CBF1/RBP-Jκ/Suppressor of Hairless/LAG-1) proteins comprise a family of transcription factors that are critical for metazoan development. As the effector components of the Notch receptor signalling pathway, they are context-dependent activators or repressors of target genes required for various cell differentiation-related decisions [7], [8]. Abnormal CSL signalling has been implicated in severe developmental defects and in several types of cancer [9], [10]. Interestingly, a number of viruses encode proteins that can hijack CSL factors to help viral replication [11].

The Notch pathway is confined to metazoa [12], but we have documented the unexpected existence of two novel CSL classes, named F1 and F2, in several species of fungi, including fission yeast as well as medically or economically relevant taxa. Notably, class F1 and F2 CSL genes are not present in the widely studied budding yeast, *Saccharomyces cerevisiae*. In all fungal genomes that harbour CSL genes, at least one representative from each fungal class was identified. The domain organization of fungal CSL proteins resembles that of their metazoan counterparts, with the notable exception of N-terminal extensions that are generally missing in animals [13]. The role of the fungal CSL family members is unclear, because fungi do not contain the Notch pathway. Interestingly, the metazoan (class M) CSL proteins have also been found to operate in a Notch-independent manner [14]–[16].

In order to gain insight into the similarities and differences between the metazoan and fungal CSL proteins, we have employed the fission yeast, *Schizosaccharomyces pombe*, as a model. We found that Cbf11 (class F1; Entrez Gene:2539560) and Cbf12 (class F2; Entrez Gene:2539119) play antagonistic roles in several cellular processes, including cell adhesion and the coordination of cell and nuclear division, the former being an important virulence trait in pathogenic fungi [17]. The opposing forces exerted by these two factors, which likely need to be well-balanced, might explain the concomitant presence of the two paralogous classes in fungi.

In this study, we conduct a more in-depth analysis of CSL protein sequences. Specifically, we explore the potential functional role(s) of the fungi-specific long N-terminal tails. We show here that these regions are likely disordered and enriched in regulatory motifs. Furthermore, in a case study of Cbf12, we demonstrate that the N-terminus negatively affects the Cbf12-dependent DNA binding activity.

## Materials and Methods

### Sequence data collection

To obtain a catalogue of the available fungal CSL family members, we have searched multiple publicly available nucleotide and protein sequence databases as described [13]. In brief, both the previously identified and novel CSL sequences were used iteratively as BLAST queries until no more new hits were found. The final searches were performed in August 2009.

The annotated gene structures for all fungal candidates were inspected manually and corrected as described [13]. The corrections consisted mostly of adjusting the predicted splicing patterns to preserve highly conserved regions. We have excluded from the study all candidates for which the sequence was incomplete (e.g., cDNA fragments), contained many apparent sequencing errors, was predicted with very low confidence, or which could not produce a robust sequence alignment (e.g., the previously reported *Cryptococcus neoformans* class F1 protein).

We have obtained a set of 33 fungal CSL sequences (including 15 novel sequences from 7 species), with 16 sequences belonging to class F1 and 17 sequences belonging to class F2. In addition, the 11 CSL family members from 8 metazoan species used previously [13] were also included in this study to represent the class M. A summary of all CSL sequences used is provided in the Table S1 and Text S1. Novel and corrected fungal CSL cDNA sequences can be found in Text S2.

### Sequence conservation and phylogenetic tree construction

Multiple sequence alignments were carried out using the ClustalX 2.0.12 algorithm with default settings [18]. The alignments were used to assign novel fungal CSL proteins to their respective class (F1 or F2), and to assist partitioning of all CSL sequences into 3 distinct regions (see the Text S3 for details): 1) the non-conserved N-terminal tail, 2) the highly conserved DNA-binding core consisting of the N-terminal Rel-homology region (RHR-N; Pfam:PF09271), the central beta-trefoil domain (BTD; Pfam:PF09270) and the βC4 linker, and 3) the less-conserved RHR-C domain (Pfam:PF01833) together with the extreme C-terminus [19]. The alignment quality scores for each position (Q-scores) generated by ClustalX were used as a proxy for sequence conservation.

An unrooted phylogenetic tree was constructed from ClustalX-aligned protein sequences using the MEGA 4.0 package [20]. All positions containing gaps were removed, and the tree was generated by the neighbour-joining method with default settings and 500 bootstrap replicates to assess node stability.

### Prediction algorithms

All algorithms were run with default settings. Protein regions of low sequence complexity (LCRs) were detected using the GBA algorithm [21]. Protein secondary structure predictions were carried out with the Phyre 0.2 integrative tool [22]. The residues predicted to form either an α-helix or a β-strand were classified as having the propensity for a well-defined secondary structure. Intrinsically disordered regions within the protein sequences were searched using Disopred3 [23] and the PONDR® VSL1 meta-predictor (Molecular Kinetics). Both approaches yielded similar outputs (data not shown), and only the PONDR® results are reported here. To identify putative phosphorylation sites, three distinct software tools were employed: KinasePhos [24], NetPhos 2.0 [25] and DISPHOS 1.3 [26]. A potential phosphorylation event was only considered when it was predicted by at least two out of the three independent predictors. The presence of PEST motifs was assessed using the epestfind tool from the EMBOSS package [27]. Only motifs classified as “potential” were considered.

### Data manipulation and statistics

Data handling was done using a set of in-house Python and R scripts. Statistical tests were performed in R. Unless stated otherwise, one-sided Wilcoxon signed-rank test with continuity correction was used at the 0.05 significance level. As the metazoan (class M) CSL N-terminal regions are in general very short, thus possibly biasing any metrics derived from them, and as this study is mostly focused on the two fungal CSL classes, the p-values for the class M N-termini statistics are not reported in the text.

### Plasmids and constructs

The plasmids and strains used for thiamine-repressible expression of N-terminally HisMyc-tagged Cbf11 and full-length Cbf12 were reported previously [17]. The corresponding Cbf12ΔN truncation mutant (aa 395–963) lacking the non-conserved N-terminus was created by removing the NdeI/SalI fragment from the original plasmid.

The fission yeast knock-in strain expressing C-terminally triple HA-tagged Cbf12 from its endogenous chromosomal locus (JB817: *h^+s^ cbf12-3HA::nat^R^*) was constructed in a wild-type background (JB32: *h^+s^*) by standard PCR-mediated one-step gene tagging using the pFA6a-3HA-natMX6 plasmid as template [28]: The forward primer (MP43: 5′-CAGTGGAATTATCTCCCATTTTATTATTTCAATACGAGACACTCTTTCATTCTGGATATAAGTGGCCTTTGGAAAGTCACCGGATCCCCGGGTTAATTAA-3′) consisted of 80 nt complementary to the 3′ end of the *cbf12* open reading frame (stop codon not included) and 20 nt complementary to the 5′ end of the 3HA-natMX6 tagging cassette (underlined). The reverse primer (MP56: 5′-GTTGTAGTAATAAATAAACACAGTAGTGCGAAAGGATATGGCAAATATGTGTAGTTGACAATAAAACCATTTTTTAAAGAGAATTCGAGCTCGTTTAAAC-3′) contained 80 nt complementary to the genomic sequence starting 80 nt downstream of the *cbf12* open reading frame (the 80 nt gap was introduced to obtain a primer with a higher melting temperature) and 20 nt complementary to the 3′ end of the 3HA-natMX6 tagging cassette (underlined). The primers were used to PCR-amplify the tagging cassette; the PCR product was gel-purified, transformed into *S. pombe* cells and nourseothricin-resistant clones, in which the cassette had been integrated by homologous recombination, were selected as described [28].

The strain expressing C-terminally double TAP-tagged Cbf12 from its endogenous chromosomal locus was constructed analogously in an auxotrophic background (JB790: *h^−^ ura4-D18 leu1-32 ade6-M216*). The MP43 forward primer and a reverse primer complementary to the region immediately downstream (i.e., without the 80 nt gap as for the MP56 primer) of the *cbf12* open reading frame (MP44: 5′-AAAACAAAAAGAGTAAAAATAAATATACTAATCCCTTGCAAAAACTTTTCAATAATAAAAAAGTAGTAAAGACAAATAATGAATTCGAGCTCGTTTAAAC-3′) were used for the amplification of the tagging cassette from the pFA6-CTAP4-natMX6 plasmid [28]. The resulting strain (JB794: *h^−^ ura4-D18 leu1-32 ade6-M216 cbf12-CTAP4::nat^R^*) was then crossed with the wild type JB32 strain to obtain a final strain without the auxotrophic markers (JB796: *h^+s^ cbf12-CTAP4::nat^R^*).

### Yeast culture and transformation

Fission yeast cells were grown according to standard procedures [29] in either rich YES or minimum MB media (Formedium). The lithium acetate method was used for transformation [30]. Expression of Cbf11, Cbf12 and Cbf12ΔN from a plasmid was regulated by the presence (repression) or absence (induction) of 15 µM thiamine in MB medium [31].

### Western blotting

Cells were harvested by centrifugation, washed with STOP buffer (150 mM NaCl, 50 mM NaF, 25 mM HEPES, 1 mM NaN_3_; pH 8) and kept at −80°C. Protein extracts were prepared by breaking the cells with glass beads in Lysis buffer 1 (25 mM HEPES, 0.1 mM EDTA, 150 mM KCl, 0.1% Triton X100, 25% glycerol, 1 M urea, 2 mM DTT, FY protease inhibitors (Serva); pH 7.6 ) or Lysis buffer 2 (for phosphoshift detection; 6 mM Na_2_HPO_4_, 4 mM NaH_2_PO_4_, 1% Nonidet P-40, 150 mM NaCl, 2 mM EDTA, 50 mM NaF, Complete protease inhibitor cocktail (Roche), 1 mM PMSF, with or w/o Phosphatase inhibitor cocktails 1 and 2 (Sigma)). Extracts were treated with the λ phosphatase (New England Biolabs) for 30 min at 30°C where required.

Proteins were separated on either a 4–12% Bis-Tris NuPAGE gradient gel (Invitrogen) or a 7.5% Tris-glycine gel, transferred on a nitrocellulose membrane and probed with either the mouse monoclonal anti-HA (H9658, Sigma) or anti-His antibody (#70796, Novagen), as appropriate. A goat-anti-mouse HRP-conjugated secondary antibody (sc-2031, Santa Cruz Biotechnology) was used for chemiluminescent detection of the tagged CSL proteins.

### LC-MS/MS analysis and phosphopeptide identification

Thirteen litres of the JB796 strain culture were grown to mid-log phase in rich YES medium. Cells were harvested by centrifugation, washed once with water and then frozen in liquid nitrogen as noodles. Cells were broken using the RM200 mortar (Retsch), lysate was prepared in Lysis buffer 2 (see above) and clarified by ultracentrifugation at 100,000 g for 40 min. Standard tandem affinity purification [32] was carried out, and the purified Cbf12 protein was eluted twice with 500 µl of Elution buffer (0.5 M NH_4_OH, 0.5 mM EDTA), precipitated with 25% TCA, washed once with ice-cold acetone containing 0.05 N HCl and once with acetone only.

Purified proteins were denatured, reduced with Tris 2-carboxyethyl phosphine, alkylated with iodoacetamide, and digested overnight at 37°C with Trypsin Gold (Promega) or Chymotrypsin (Princeton Separations) after diluting to 2 M urea with 50 mM Tris pH 8.5. The resulting peptides were subjected to 2D LC-MS/MS (MudPIT) on a Thermo LTQ as previously detailed [33], [34]. Thermo RAW files were converted to MZML files using Scansifter (software developed in-house at the Vanderbilt University Medical Center) and spectra with fewer than 20 peaks were excluded from analysis. The *S. pombe* database (http://www.sanger.ac.uk, October 2009) was searched with the Myrimatch algorithm [35] v1.6.33 on a high performance computing cluster (Advanced Computing Center for Research & Education at Vanderbilt University). We added contaminant proteins (e.g., keratin, IgG) to the complete *S. pombe* database and reversed and concatenated all sequences to allow estimation of false discovery rates (10,186 total entries). Myrimatch search parameters were as follows: strict tryptic cleavage; modification of methionine (oxidation, dynamic modification, +16 Da), S/T/Y (phosphorylation, dynamic modification, +80 Da) and cysteine (carboxamidomethylation, static modification, +57 Da) were allowed; precursor ions were required to be within 0.6 *m/z* of the peptide monoisotopic mass; fragment ions were required to fall within 0.5 *m/z* of the expected monoisotopic mass. IDPicker [36], [37] v2.6.165 was used to filter peptide matches with the following parameters: max. FDR per result 0.05, max. ambiguous IDs per result 2, min. peptide length per result 5, min. distinct peptides per protein 2, min. additional peptides per protein group 2, minimum number of spectra per protein 5, indistinct modifications M 15.994 Da, C 57.05 Da and distinct modifications S/T/Y 80 Da. Actual FDR for Cbf12 analysis was 0.5%. Spectra indicative of phosphopeptides were manually inspected in SeeMS and a related program, called PTMDigger, software developed in-house (Surendra Dasari, Matthew Chambers, and David Tabb, Vanderbilt University Medical Center) and filtered according to the following criteria: (1) exhibit a prominent (often base) 98 Da (H_3_PO_4_) neutral loss peak at the MS2 level and (2) b and y ion intensities >20% of the neutral loss peak (3) contained two or more sequential fragments (b and/or y) bracketing the phosphorylation site(s). Phosphorylation sites were assigned based on the presence of sequential fragment ions surrounding the modification; if these ions were missing, the phosphorylation site(s) were assigned to multiple sites ambiguously.

### Electrophoretic mobility shift assay

The analysis of DNA binding by fission yeast CSL proteins was described in detail previously [17]. Briefly, native extracts were prepared from CSL double knock-out cells expressing tagged Cbf11, Cbf12 or Cbf12ΔN from a plasmid, and binding to radiolabelled double-stranded DNA probes, which contain either a canonical metazoan CSL binding site (metazoan promoter-derived probe ‘RBP’ [17]; probes ‘ste6’: 5′-CGATTACATCCGTGGGAAAAAACATTTGTT-3′ and ‘c1450.16c’: 5′-ACAAATGTTTTTTCCCACGGATGTAATCGT-3′ derived from fission yeast *ste6* and SPCC1450.16c gene promoters, respectively; the CSL binding site is underlined), or a mutated CSL site (probe ‘DEL’ [17]) in the presence of excess carrier DNA was detected as a slow-migrating band on a large native 5% polyacrylamide gel. Competition experiments were performed with 100-fold excess of the respective unlabelled probes.

## Results

### Novel fungal CSL proteins

**Figure 1:**
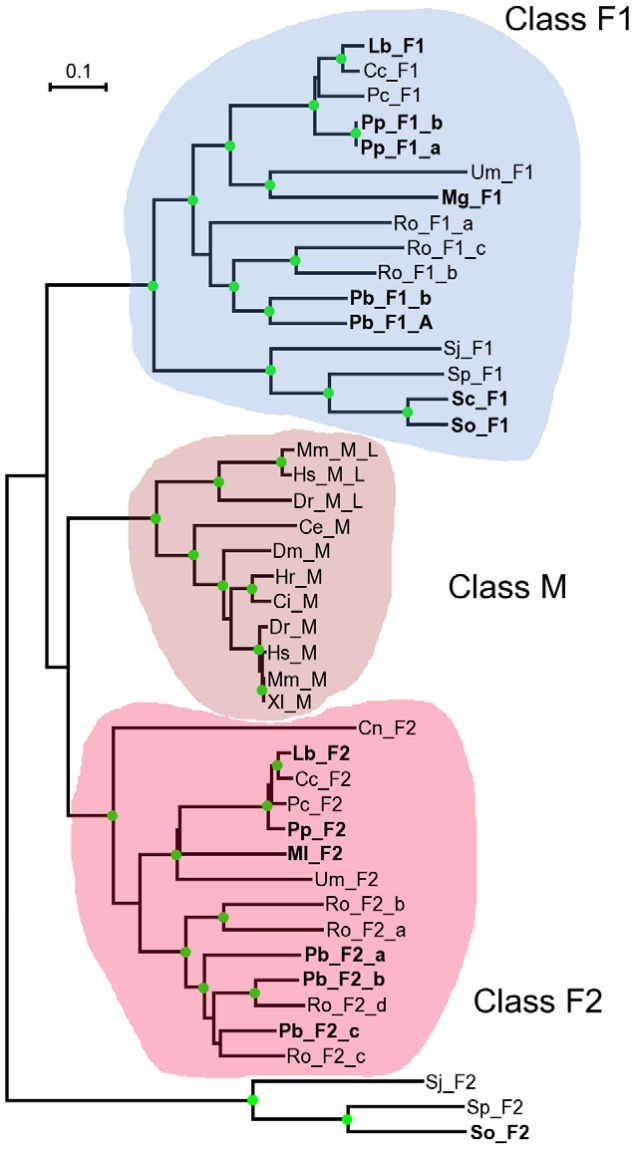
Phylogenetic distribution of CSL proteins used in this study. An unrooted neighbour-joining phylogenetic tree of all CSL proteins analysed in this study. Novel CSL sequences (labelled in bold) follow the taxonomical distribution of those published previously [13]. *Schizosaccharomyces pombe* (Sp), *S. octosporus* (So), *S. japonicus* (Sj) and *S. cryophilus* (Sc) belong to *Taphrinomycotina*, the basal subphyllum of ascomycetes. *Coprinus cinereus* (Cc), *Cryptococcus neoformans* (Cn), *Laccaria bicolor* (Lb), *Malassezia globosa* (Mg), *Melampsora laricis-populina* (Ml), *Phanerochaete chrysosporium* (Pc), *Ustilago maydis* (Um) and *Postia placenta* (Pp) are basidiomycetes. *Rhizopus oryzae* (Ro) and *Phycomyces blakesleeanus* (Pb) are zygomycetes. Representative metazoan CSL sequences are from human (Hs), mouse (Mm), zebrafish (Dr), *Xenopus laevis* (Xl), *Ciona intestinalis* (Ci), *Halocynthia roretzi* (Hr), fruit fly (Dm) and *Caenorhabditis elegans* (Ce). Paralogs are denoted by letter suffixes (see Table S1 for more information). The three CSL classes are indicated by coloured background (F1 – blue; F2 – red, M – brown). The class F2 fission yeast branch position is of low confidence and therefore not shaded. Green circles at nodes indicate ≥90% bootstrap stability. The scale bar indicates the number of amino acid substitutions per site.

We have previously shown that the CSL transcription factor family, which plays a key role in the Notch signalling pathway critical for animal development, is not confined to metazoa but additionally exists in fungi, unlike most of the Notch pathway components. We have previously identified 19 CSL representatives in 7 species of fungi forming two distinct, fungi-specific classes, designated F1 and F2. Both classes are present in the genome of each of these 7 species [13]. Here we extended this analysis by searching the wealth of new fungal sequence data that recently became publicly available. We collected 15 novel high-quality CSL protein sequences from a further 7 species. The new findings are in agreement with the phylogenetic distribution reported in our initial study [13], with no evidence for CSL proteins in ascomyces beyond the *Taphrinomycotina* basal branch (e.g., fission yeast). Our final fungal set contained 33 unique CSL proteins (16 class F1, 17 class F2); three fungal species were only represented by a single CSL protein, because the other paralog did not pass our sequence quality control criteria. For *Malassezia globosa*, only a single CSL protein (class F1) was found in the GenBank database. For comparison, 11 selected metazoan CSL proteins from 8 species ranging from *C. elegans* to human, were also used in this study (Figure 1).

### CSL domain composition and conservation

**Figure 2:**
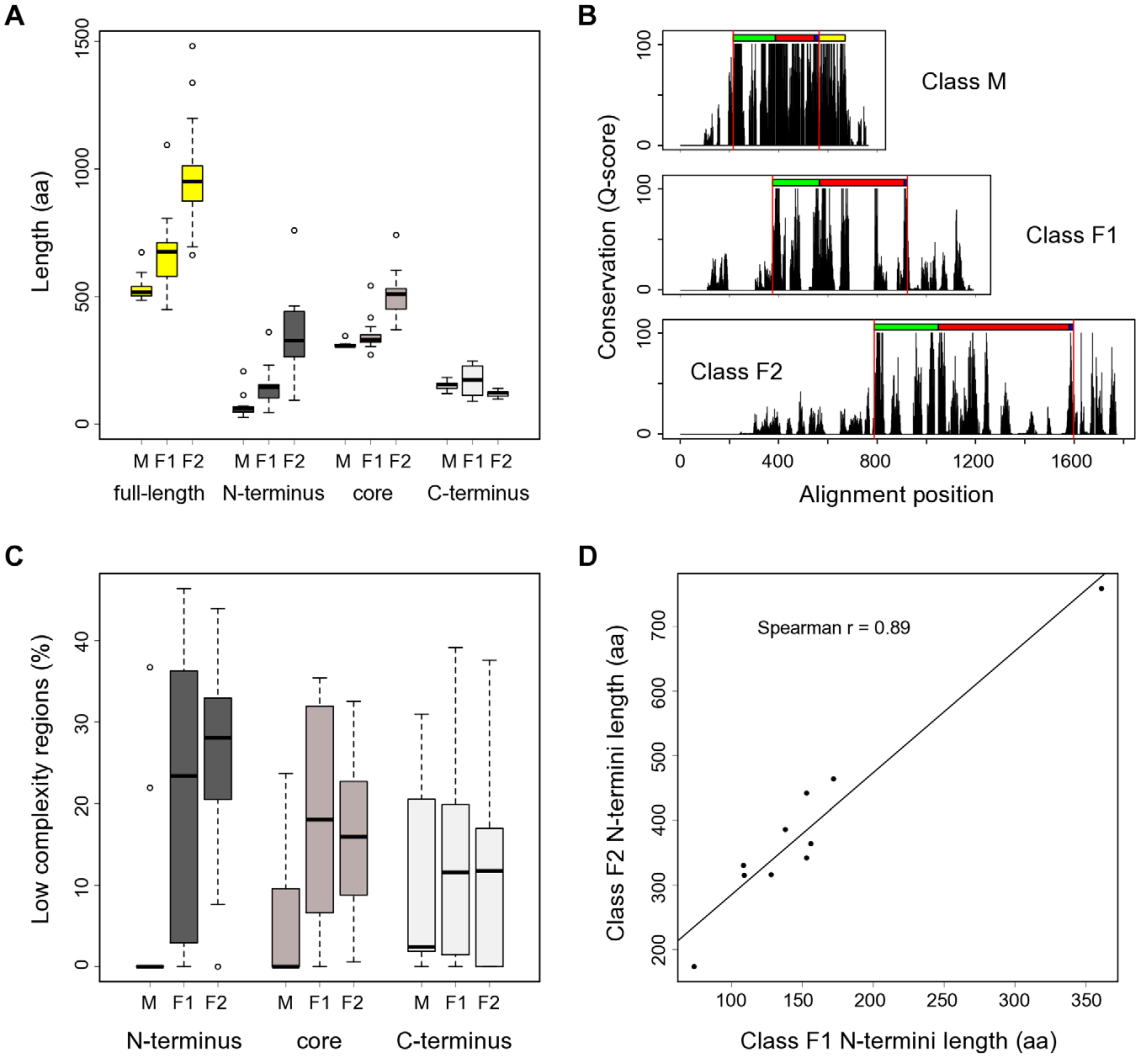
CSL protein length, organization and conservation. (**A**) Fungal CSL proteins contain notable extensions in their N-termini (class F1, F2) and core (class F2). Whisker plots showing size distributions of the CSL proteins used in this study both for full-length sequences and their respective N-terminal, core, and C-terminal regions. M (n = 11), F1 (n = 16) and F2 (n = 17) denote the three distinct classes within the CSL family. (**B**) Sequence conservation profiles for the individual CSL classes (based on a gapped ClustalX protein alignment) show marked differences between the N-terminal, core, and C-terminal regions. The known domain composition is indicated above each profile: RHR-N (green), BTD (red), βC4 linker (blue), RHR-C (yellow; divergent in fungi). Red vertical lines show the partitioning into the 3 regions described in the main text. (**C**) Distribution of low-complexity regions across the CSL protein sequences and classes reveals a higher abundance of LCRs in the fungal homologs. The percentages of sequence scored as having a low complexity are shown. Note that the results for N-termini of class M are affected by the very short length of that region in this class. (**D**) The length ratios of N-termini between the F1 and F2 classes are conserved in all species tested. Each data point represents a fungal species; the coordinates are the corresponding class F1 and F2 paralog N-termini lengths, respectively. Mean values were plotted for species with multiple paralogs per class. Only species with both F1 and F2 representatives present in our dataset were included.

The crystal structures of metazoan (class M) CSLs revealed that these proteins have a unique fold consisting of two Rel-like domains (RHR-N and RHR-C) with an intervening beta-trefoil domain. These domains are further flanked by short N- and C-terminal extensions of low sequence conservation and unknown fold [38], [39]. Based on the crystal structure data and on our previous sequence analyses [13], we partitioned all CSL sequences in this study into 3 regions corresponding to the non-conserved N-terminal extension, the highly conserved DNA-binding core, and the RHR-C domain with the C-terminal tail (Figure 2A,B; Materials and Methods).

As noted earlier, the proteins in both fungal classes are typically much longer than their mammalian counterparts. This property is largely attributable to the presence of a long insertion in the BTD of most class F2 proteins, and, even more importantly, to the presence of unusually long extensions at the class F1 and F2 N-termini [13]. These long N-terminal tails are devoid of any known domains (data not shown) and on average comprise 21.4% (F1) and 34.3% (F2) of the whole protein length. By contrast, the average class M amino tail represents just 12.8% of the protein (Figure 2A).

The amino acid sequence of the N-terminal regions is poorly conserved (Figure 2B) and is highly divergent even amongst closely related species (Text S3). Visual inspection of the fungal N-termini revealed frequent homooligomeric stretches, and a more rigorous analysis confirmed a trend for increased incidence of low-complexity regions compared with the core and C-termini (Figure 2C; statistically significant for class F1 C-termini, and class F2 core and C-termini, p≤0.014). As there are few experimental data available for the fungal CSL proteins, we considered the possibility that the N-termini are artefacts of automatic genome annotation and do not encode amino acids. However, the corresponding regions of CSL genes are transcribed in fission yeast [40], and proteins show the predicted size when expressed as chromosomally tagged fusions [17] (see below and data not shown). Strikingly, the per species class F1/F2 N-termini length ratio is highly conserved in fungi (Figure 2D, Spearman correlation r = 0.88, p = 0.0006). Furthermore, the 5′ regions of fungal CSL mRNAs show no conserved structural motifs that might suggest any function of these sequences at the RNA level (data not shown). Based on these findings, we hypothesized that the extended N-termini of fungal CSL proteins are expressed and functionally important, despite their highly divergent sequence.

### CSL N-termini are likely intrinsically disordered

**Figure 3:**
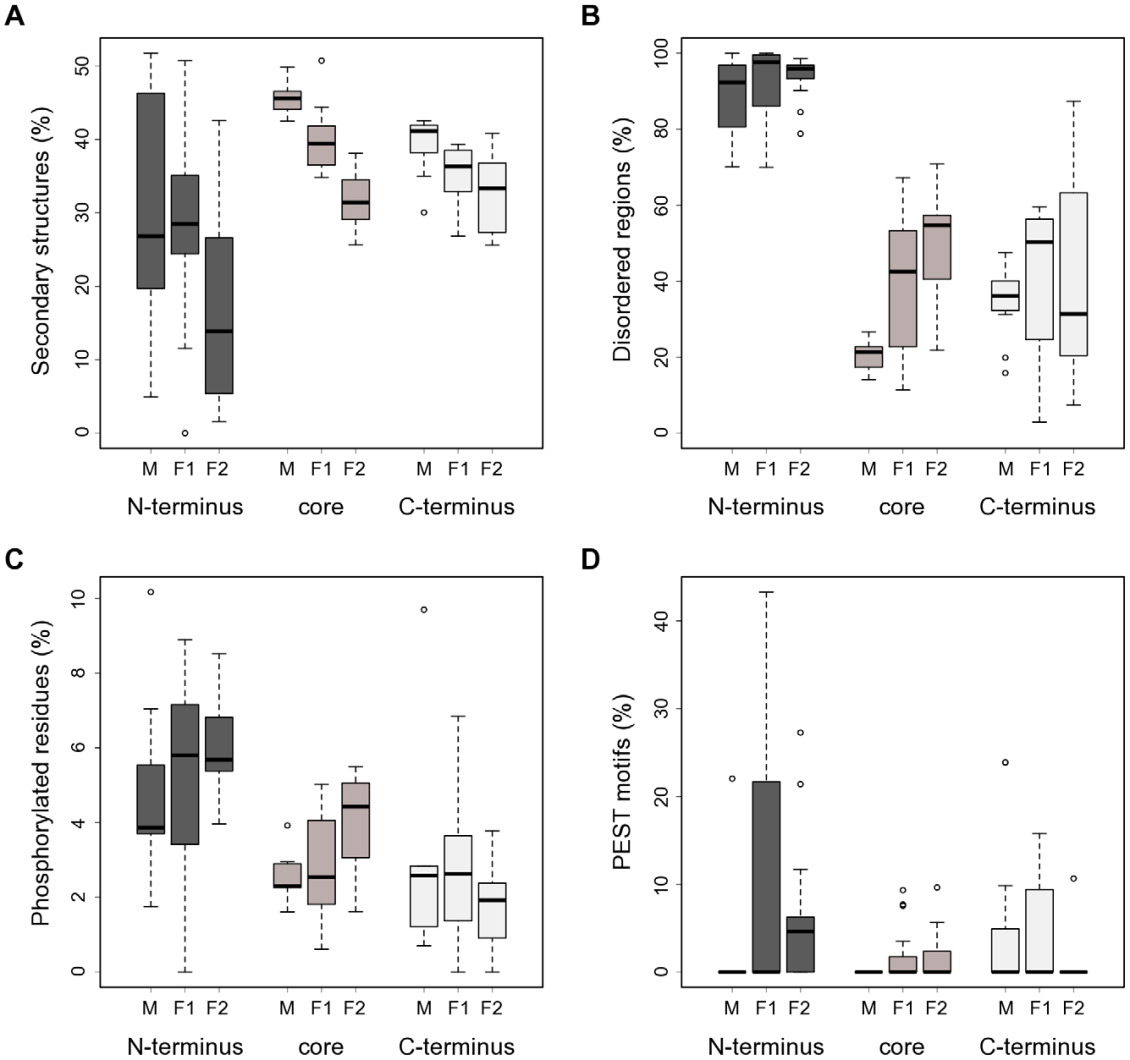
CSL N-termini are predicted to be disordered and enriched in potential regulatory motifs. (**A**) The N-termini of CSL proteins show a marked depletion of predicted α-helices and β-strands, which is most striking for class F2. The percentages of residues predicted to form secondary structures are shown for the respective N-terminal, core, and C-terminal regions of the CSL proteins used in this study. Note that the results for N-termini of class M are affected by the very short length of that region in this class. M (n = 11), F1 (n = 16) and F2 (n = 17) denote the three distinct classes within the CSL family. (**B**) The N-termini of CSL proteins are likely intrinsically disordered, as judged by the output of the PONDR® meta-predictor. The percentages of residues predicted to be disordered per CSL region and class are shown. (**C**) The CSL N-terminal regions are enriched for potential phosphorylation sites compared to the rest of the protein. The percentages of amino acids predicted by at least 2 out of 3 independent algorithms to be phosphorylated in the indicated regions are shown for all CSL classes. (**D**) Potential PEST motifs are enriched in the N-termini of fungal CSL proteins. The fractions of sequence conforming to the PEST motif definition in each region and CSL class are shown.

As mentioned above, Pfam analysis [41] did not identify any known domains in the amino-terminal portions of CSL proteins. Therefore, we obtained predictions of secondary structures for all CSL proteins in our set to provide information towards the function of the N-termini. The prediction results were in good agreement with the published *C. elegans* LAG1 crystal structure [19] (Entrez Structure:1TTU), showing a high prevalence of β-strands and several α-helices in the portion of the protein covered by the crystallographic study (data not shown). Notably, we detected a marked depletion of well-defined secondary structure elements (α-helices and β-strands) in the N-termini of all three CSL classes compared to the other two protein regions (Figure 3A; statistically significant for the F1 and F2 classes, p≤0.015).

The importance of intrinsically disordered regions has been recognized for processes such as signal transduction and transcription [3]. The predicted lack of secondary structure together with the abundance of LCRs in the CSL N-termini might reflect that these regions are intrinsically disordered. A number of bioinformatic tools are available now to detect such disordered regions (e.g., [3], [42]). As suspected, analysis by the PONDR® disorder meta-predictor showed a striking degree of intrinsic disorder in the N-terminal tails of CSL (Figure 3B). On average, 88.6%, 91.9% and 94.1% of the N-terminal sequence length was predicted to be disordered in class M, F1 and F2, respectively.

### Regulatory motifs in CSL N-termini

Protein phosphorylation is a post-translational modification well-known for the regulation of DNA-binding factors [1], [2]. Interestingly, intrinsic disorder has been associated with the presence of phosphorylation target sites [26], [43]. Furthermore, the Composition profiler tool [44] identified a significant overrepresentation of serine residues in CSL N-terminal regions (two-sample t-test with Bonferroni correction, p<0.0001; SwissProt 51 dataset used as reference), raising the possibility that the tails might serve as kinase substrates. To explore this possibility, we ran multiple phosphorylation prediction algorithms and constructed consensus profiles of putative phosphorylation sites for our CSL dataset (Materials and Methods). The analysis of these profiles confirmed a statistically significant kinase target site enrichment (over the core and C-terminal regions) in the N-termini of CSL (Figure 3C; p≤0.005). Thus, the extended amino-terminal tails could potentially mediate regulation of the CSL transcription factors via a protein kinase.

Another hallmark of the CSL N-termini identified by the analysis above was a strong enrichment in prolines (p<0.0001). PEST motifs, amino acid sequences rich in proline, glutamic acid, serine and threonine, are condition-specific protein degradation signals [45]. Notably, PEST motifs are preferentially situated in disordered regions and correlate with regulatory biological processes [46]. Therefore, we searched for potential PEST motifs in our CSL dataset. The results are summarized in Figure 3D and, indeed, show a statistically significant overrepresentation of PEST motifs in the N-termini of class F2 (vs. core and C-termini, p≤0.007) and, weakly, in the N-termini of class F1 (vs. core, p = 0.038). Taken together, the N-terminal regions of fungal CSL proteins are enriched for two important types of regulatory sequences, kinase target sites and PEST motifs. These data raise the possibility that the N-terminal regions play important roles in CSL regulation.

### Class F2 CSL N-terminus affects binding to DNA in fission yeast

**Figure 4:**
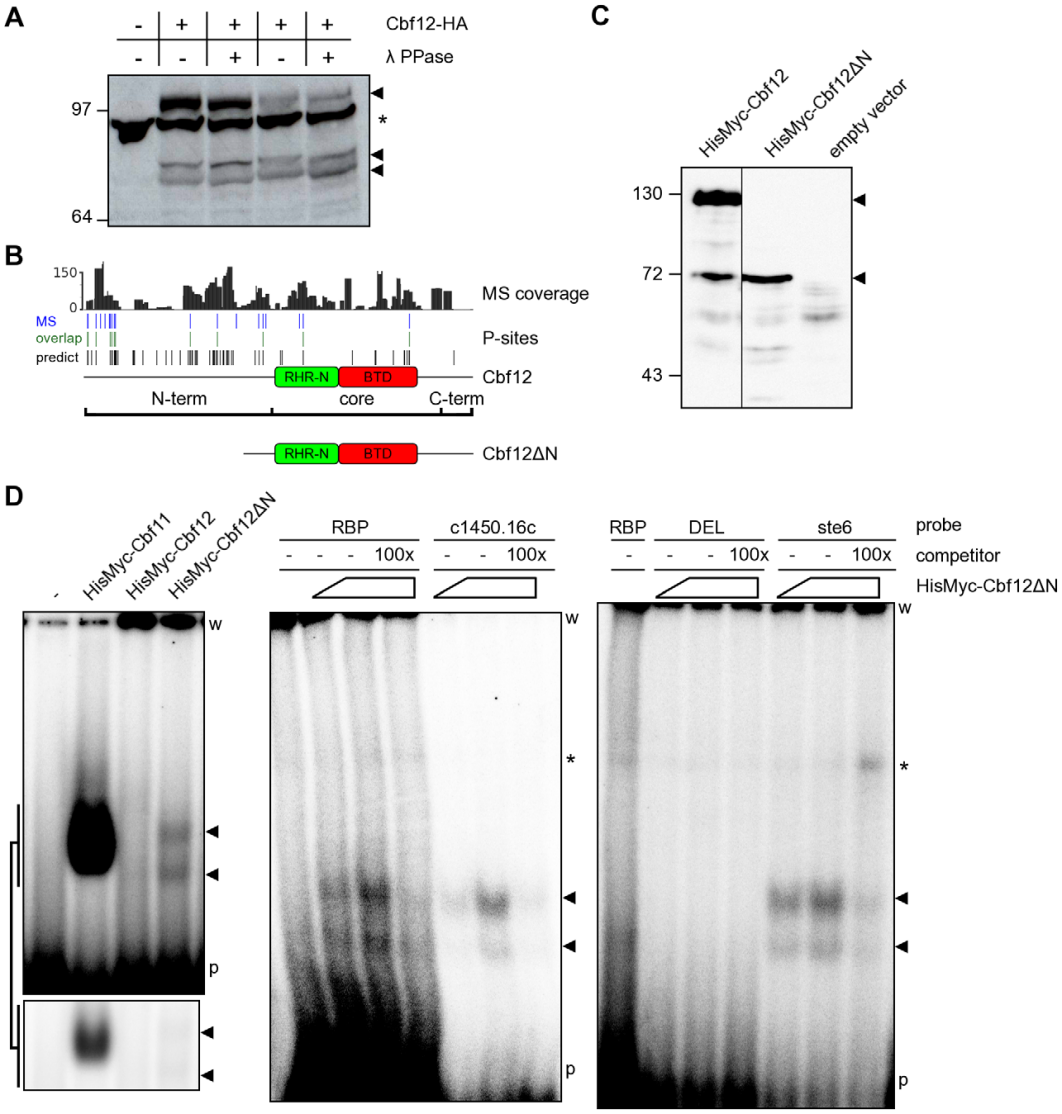
DNA binding is affected by the N-terminus of Cbf12. (**A**) Phosphatase treatment of the C-terminally HA-tagged Cbf12 protein results in a higher gel migration speed, indicating that Cbf12 (arrowheads) is phosphorylated. Two independent samples, lanes 2–3 and 4–5, respectively, are shown in this western blot. Note the presence of smaller proteolytic products. The asterisk denotes a cross-reacting, unrelated band. (**B**) Analysis of Cbf12 phosphorylation sites by mass spectrometry. The plots show (from top to bottom) Cbf12 coverage depth, the positions of 19 experimentally identified phosphorylation sites, and their overlap (12 sites) with 61 predicted phosphorylation sites. Schematic representations of full-length and truncated Cbf12 proteins are also shown. (**C**) Cbf12ΔN, a truncation mutant lacking most of the N-terminal region (amino acids 1–394) was constructed and its expression verified by western blot. Note that full-length Cbf12 in lane 1 is also present as multiple species when tagged at the N-terminus (cf. Figure 4A). (**D**) Electrophoretic mobility shift assay: (left panel) a DNA-binding activity recognizing the CSL consensus site on a radiolabelled ‘RBP’ DNA probe is present in cell extract containing Cbf12ΔN (arrowheads), but not in extract with full-length Cbf12. The Cbf11 DNA-binding activity and a control lane with extract from cells without any plasmids (‘-’) are also shown for comparison. The bottom inset shows a shorter exposure of the gel area with Cbf11/Cbf12ΔN bands. (middle and right panels) Increasing amounts of cell extract containing Cbf12ΔN were incubated with various probes containing the CSL binding site (‘RBP’, ‘c1450.16c’, ‘ste6’) or a mutated site (‘DEL’) with or without unlabelled competitor probes. The asterisk denotes a non-specific band, which is also present in the no-extract control lanes. ‘w’ – wells; ‘p’ – unbound probe.

The bioinformatic analyses conducted so far generated several hypotheses that could be tested experimentally. We exploited the data and resources available for the fission yeast CSL proteins Cbf11 and Cbf12, members of class F1 and F2, respectively, to validate some of the predictions [17]. One such prediction was that fungal CSL proteins are preferentially phosphorylated at their N-termini. To test this possibility, we first expressed C-terminally HA-tagged Cbf12 from its endogenous locus and under the control of its natural promoter, prepared whole-cell extracts and assayed for phosphorylation using λ phosphatase. Any removed phosphate groups would decrease the molecular weight of Cbf12 and thus increase its migration in a polyacrylamide gel. As shown in Figure 4A, there was indeed a downshift of Cbf12 in phosphatase-treated lanes, confirming that Cbf12 is a phosphoprotein. Even though the downshift was rather modest, given the masses of Cbf12-HA (∼114 kDa) and that of a phosphate group (80 Da), the fact that we were able to see a downshift for such a large protein indicates that Cbf12 is actually phosphorylated at multiple sites.

To obtain more refined information on Cbf12 phosphorylation, we conducted large-scale purification of TAP-tagged Cbf12 and subjected the purified protein to analysis by mass spectrometry. We achieved 82.7% overall coverage of the Cbf12 sequence with the average coverage depth being 51.1-fold, 44.7 -fold and 30.5-fold for the N-terminal, core and C-terminal region, respectively. In this high-coverage dataset we have identified 16, 3 and 0 phosphorylated sites comprising 3.5%, 0.8% and 0% of the N-terminal, core and C-terminal region (Figure 4B and Table S2), respectively, confirming our hypothesis that Cbf12 is preferentially phosphorylated at its N-terminal region.

The gel in Figure 4A also showed that Cbf12 is present in the extract as multiple species of different length, suggesting that the protein is proteolytically processed in the cell. It is unlikely that these multiple isoforms occur due to the usage of alternative transcription start sites, because we could still detect multiple bands when Cbf12 was tagged at the other end and expressed from a plasmid as a HisMyc N-terminal fusion (Figure 4C). Furthermore, overexposures of the blot from Figure 4A revealed yet additional, weaker bands, suggesting that Cbf12 proteolysis is taking place. By contrast, no proteolysis could be detected for the paralogous Cbf11 protein under the same conditions (data not shown). In our previous study we have detected sequence-specific DNA binding for Cbf11, but not for Cbf12 [17]. This finding is puzzling given the fact that the DNA-binding regions are well conserved in both fission yeast CSL proteins [13]. The data presented here indicate that Cbf12 may undergo proteolysis. From comparison of the C- and N-terminally tagged Cbf12 data, the major cleavage site seems to be located in the core-proximal part of the PEST motif-containing N-terminus of Cbf12. In order to test the functional significance for the shorter Cbf12 fragments we observed (Figure 4A), we constructed a truncated version (Cbf12ΔN) that lacked most of the N-terminal region (amino acids 1–394; Figure 4B) and only retained 6 out of the 19 phosphorylation sites identified by mass spectrometry. We hypothesized that the presence of the N-terminal tail regulates the ability of Cbf12 to bind to DNA. To test this idea we compared the affinity of Cbf11, full-length Cbf12 and Cbf12ΔN, respectively, to a metazoan promoter-derived DNA probe (probe ‘RBP’; [17]) containing the canonical CSL response element (Figure 4D, left panel). Notably, while Cbf12 displayed no detectable binding, a clear and specific DNA binding activity was present in the sample containing Cbf12ΔN and this activity was distinct from that of Cbf11. Moreover, the Cbf12ΔN-dependent affinity was also detected when additional probes were used, derived from fission yeast promoters containing the canonical CSL binding site (‘ste6’, ‘c1450.16c’), whereas no activity was detected with a probe containing a mutated CSL binding site (‘DEL’; [17]) (Figure 4D, middle and right panels). Finally, the binding activity could be specifically competed with an excess of the respective unlabelled probes. Thus, we demonstrate here a Cbf12-dependent DNA binding activity that recognizes the same CSL target site as does the paralogous Cbf11 protein; this DNA-binding activity is inhibited by the extended and divergent N-terminus of Cbf12.

## Discussion

We report in this study that fungal CSL proteins contain large regions of computationally predicted intrinsic disorder in their extended N-termini, and that these regions are enriched in two types of regulatory elements: phosphorylation sites and PEST motifs. We also provide experimental evidence that Cbf12, the fission yeast class F2 CSL protein, is phosphorylated with the majority of phosphorylated sites being located in the N-terminal region of Cbf12. Moreover, our data suggest that Cbf12 undergoes regulated proteolysis, and that the removal of its N-terminal tail enables the protein to bind to DNA, a property not observed for the full-length protein.

Despite their low sequence complexity and low degree of evolutionary conservation, the long N-termini of fungal CSL proteins show conserved features, which suggest that these regions are important for CSL function. Many fungi with CSL proteins are simple organisms with short generation times and small, streamlined genomes [47]–[49]. The CSL family likely originated in the last common ancestor of fungi and animals [13], and it would be expected that large non-functional protein sequences would have been removed over time by natural selection [50]. The finding that these tails are always present and that there is a striking relationship between the N-termini lengths of class F1 and F2 paralogs in all species in our dataset (Figure 2D) strongly argues for functional significance.

These N-terminal tails are rich in potential kinase target sites and are likely disordered, which is expected to broaden the spectrum of proteins that can access them [6]. This property could make the N-termini ideal platforms for integrating inputs from multiple signal transduction pathways. Consistent with this idea, we have here experimentally identified 16 phosphorylation sites throughout the N-terminal region of Cbf12 (including a site reported in a recent proteomics study [51]).

Both fission yeast CSL paralogs are involved in the same biological processes (adhesion, cell-cycle regulation, ploidy maintenance), but their roles in these processes seem to be antagonistic. The relative levels of both Cbf11 and Cbf12 need to be finely tuned ([17] and our unpublished data). PEST motifs, enriched in particular in the N-termini of class F2 sequences, are closely linked to protein phosphorylation. They represent regulatory motifs that can direct either protein degradation by the proteasome or its cleavage by the calpain or caspase types of proteases [45], [52], [53]. The activity of PEST motifs is often condition-specific as, for example, a PEST region might only become exposed, and thus functional, upon a certain trigger event. Phosphorylation (either in the PEST motif itself or elsewhere in the protein) is a prominent factor in the regulation of PEST activity [45]. We have noted that the overexpression of Cbf12 is toxic in fission yeast [17]. Disordered proteins have been found to be tightly regulated [54], [55]. Thus degradation by the 26S proteasome, possibly mediated by a PEST motif and phosphorylation, could constitute a regulatory mechanism whereby a cell keeps Cbf12 amounts at appropriate levels in different conditions. In addition to such regulated degradation, a more targeted proteolytic step could be employed to allow for binding of Cbf12 to DNA as and when required. Dual-mode proteolysis by the proteasome and calpain has been described for example for the murine YY1 and human NF-κB transcription factors, which notably affects the DNA-binding activity of the latter [56], [57]. In a CSL double deletion background, the Cbf12ΔN truncation mutant displays a clear DNA binding activity, which is not the case for full-length Cbf12 (Figure 4C). The Cbf12ΔN-dependent binding of the ‘RBP’ probe is weaker than that of Cbf11, which might reflect target site preferences of the individual CSL factors. Alternatively, the truncation of the N-terminus in Cbf12ΔN may not exactly replicate the conditions of the hypothetical active state in Cbf12. While it cannot be ruled out from the current data that the observed DNA binding activity is only indirectly caused by Cbf12ΔN, we consider this possibility less likely for the following reasons: 1) recombinant Cbf11 can bind to the ‘RBP’ probe directly, and the residues required for sequence-specific DNA binding are conserved in the primary sequence of Cbf12 [17]; 2) we have never detected any binding to the ‘RBP’ probe in extracts from *Δcbf11* or *Δcbf11 Δcbf12* cells under multiple experimental conditions, which suggests that no other *S. pombe* protein can bind to the ‘RBP’ probe (MPř, MPt, PF, FP, unpublished observations). The presence of the N-terminus thus seems to inhibit the ability of Cbf12 to interact with DNA, perhaps by imposing a sterical obstruction, or by affecting the binding site conformation or contacts with interaction partners. The situation is obviously more complex in a wild-type background where both full-length and truncated Cbf12 isoforms are found together (Figure 4A), and other factors such as subcellular localization, or competition with Cbf11 for DNA target sites, may play a role. Nevertheless, the proposed regulation of Cbf12 activity by its N-terminal region may represent an important means by which the fission yeast cell strikes a balance between the class F1 and class F2 paralog activities. This hypothesis remains to be fully tested experimentally, together with exploring the generality of these observations for other CSL-positive fungal species.

It will be interesting to see how the data from fungal systems apply to the CSL family members of class M. To our knowledge, there is little evidence for post-translational modifications of metazoan CSL proteins. It has been found that the human CBF1 is acetylated on K266 and phosphorylated on S230, which are located in the DNA-binding BTD. However, both pieces of evidence come from large-scale proteomics studies with limited biological conclusions for CBF1 [58], [59]. The N-termini of vertebrate CSL are short (Figure 2A), but the amino tails of the fruit fly and nematode homologs are 114 and 207 aa in length, respectively, and both have a high degree of predicted disorder. The significance of this observation is unclear, but it is possible that in invertebrates the CSL N-termini affect protein stability and/or activity in a manner analogous to the mechanism proposed here for fungi. Interestingly, it has been reported that the drosophila CSL homolog is targeted for degradation upon decrease in Notch signalling. This process is mediated by ubiquitinylation of the CSL protein, although the proteolytic enzyme involved is not the 26S proteasome [60].

Interestingly, a prominent role for intrinsic disorder has recently been suggested for class M RHR-C domain, which apparently undergoes coupled folding/binding during the interaction with Notch ankyrin repeats [61]. The RHR-C sequence is rather divergent in the fungal family members, which might reflect the absence of Notch in these organisms. Also, our computational predictions did not provide support for strong enrichment of intrinsic disorder in the fungal C-termini.

The data and conclusions on class F1 and F2 CSL proteins presented here should be useful even if the proposed regulatory mechanism proves to be fungi-specific. Some of the species discussed in this paper are relevant to human disease. For example, *C. neoformans* may cause meningitis in immunocompromised individuals [62], *Malassezia globosa* has been associated with skin diseases [63], and *Rhizopus oryzae* is an opportunistic human pathogen causing potentially fatal mucormycoses [64]. Moreover, other taxons are plant parasites (*U. maydis*; [48]), cause decay of wood (*Postia placenta*; [65]), or can degrade pesticides and toxic waste (*Phanerochaete chrysosporium*; [66]), making them important from economic and technological points of view. Notably, the fission yeast CSL proteins have been implicated in regulating cell adhesion, which plays a crucial role in host-pathogen interaction and is an important virulence trait [67]. It remains to be seen whether this role is shared by CSL proteins in other fungal species. If this proves to be the case, and if the proposed regulatory mechanisms controlling the balance between the class F1 and F2 opposing activities are also generally applicable, considerable benefits might come from finding the upstream regulators, and designing specific kinase and/or protease inhibitors targeting the pathways acting upon fungal CSL proteins.

## Supporting Information

Table S1 Summary of CSL sequences used (HTML).
(HTML)

Table S2 Cbf12 phosphopeptides identified by mass spectrometry (DOC).
(DOC)

Text S1 Sequences of CSL proteins used (TXT).
(TXT)

Text S2 New and corrected CSL cDNA sequences (RTF).
(RTF)

Text S3 Multiple sequence alignment used for CSL sequence partitioning (TXT).
(TXT)
