# Supplementary material for: N-Termini of Fungal CSL Transcription Factors Are Disordered, Enriched in Regulatory Motifs and Inhibit DNA Binding in Fission Yeast
Source: PLoS One. 2011 Aug 12;6(8):e23650. doi: 10.1371/journal.pone.0023650 (PMC3155561; doi:10.1371/journal.pone.0023650)
Supplement: Table S1 — Summary of CSL sequences used (HTML). (HTML) [file pone.0023650.s001.html]

|  |  |  |  |  |  |
| --- | --- | --- | --- | --- | --- |
| **Table S1 - Summary of CSL sequences used** |  |  |  |  |  |
| **Organism** | **Accession no.** | **Paralog ID** | **CSL class** | **DBase** | **Notes** |
| Caenorhabditis elegans | AAB03859 |  | M | GenBank (http://www.ncbi.nlm.nih.gov/sites/entrez?db=Protein&itool=toolbar) |  |
| Ciona intestinalis | O76808 |  | M | GenBank (http://www.ncbi.nlm.nih.gov/sites/entrez?db=Protein&itool=toolbar) |  |
| Danio rerio | NP\_942579 |  | M | GenBank (http://www.ncbi.nlm.nih.gov/sites/entrez?db=Protein&itool=toolbar) |  |
| Danio rerio | XP\_697075 | L | M | GenBank (http://www.ncbi.nlm.nih.gov/sites/entrez?db=Protein&itool=toolbar) |  |
| Drosophila melanogaster | P28159 |  | M | GenBank (http://www.ncbi.nlm.nih.gov/sites/entrez?db=Protein&itool=toolbar) |  |
| Halocynthia roretzi | BAA20141 |  | M | GenBank (http://www.ncbi.nlm.nih.gov/sites/entrez?db=Protein&itool=toolbar) |  |
| Homo sapiens | AAH64976 |  | M | GenBank (http://www.ncbi.nlm.nih.gov/sites/entrez?db=Protein&itool=toolbar) |  |
| Homo sapiens | NP\_055091 | L | M | GenBank (http://www.ncbi.nlm.nih.gov/sites/entrez?db=Protein&itool=toolbar) |  |
| Mus musculus | NP\_033061 |  | M | GenBank (http://www.ncbi.nlm.nih.gov/sites/entrez?db=Protein&itool=toolbar) |  |
| Mus musculus | NP\_033062 | L | M | GenBank (http://www.ncbi.nlm.nih.gov/sites/entrez?db=Protein&itool=toolbar) |  |
| Xenopus laevis | Q91880 |  | M | GenBank (http://www.ncbi.nlm.nih.gov/sites/entrez?db=Protein&itool=toolbar) |  |
| Coprinus cinereus | CC1G\_13688 |  | F1 | Broad Institute (http://www.broad.mit.edu/annotation/genome/coprinus\_cinereus/) |  |
| Coprinus cinereus | CC1G\_03194 |  | F2 | Broad Institute (http://www.broad.mit.edu/annotation/genome/coprinus\_cinereus/) |  |
| Cryptococcus neoformans | CNAG\_01173 |  | F2 | Broad Institute (http://www.broadinstitute.org/annotation/genome/cryptococcus\_neoformans/) | intron 2 manually included into cDNA |
| Laccaria bicolor | EU2.LBSCF0001G04170 |  | F1 | DOE Joint Genome Institute (http://genome.jgi-psf.org/Lacbi1/Lacbi1.home.html) |  |
| Laccaria bicolor | EU2.LBSCF0005G03540 |  | F2 | DOE Joint Genome Institute (http://genome.jgi-psf.org/Lacbi1/Lacbi1.home.html) | N-terminus manually truncated based on homology with C. cinereus and the Kozak sequence |
| Malassezia globosa | XP\_001730963 |  | F1 | GenBank (http://www.ncbi.nlm.nih.gov/sites/entrez?db=Protein&itool=toolbar) |  |
| Melampsora laricis-populina | ESTEXT\_FGENESH2\_PG.C\_150143 |  | F2 | DOE Joint Genome Institute (http://genomeportal.jgi-psf.org/Mellp1/Mellp1.home.html) |  |
| Phanerochaete chrysosporium | FGENESH1\_PG.C\_SCAFFOLD\_11000203 |  | F1 | DOE Joint Genome Institute (http://genome.jgi-psf.org/Phchr1/Phchr1.home.html) | N-terminus manually truncated, based on aa homology and exon/intron structure of C. cinereus and L. bicolor F1 |
| Phanerochaete chrysosporium | Pc\_CSL2 |  | F2 | DOE Joint Genome Institute (http://genome.jgi-psf.org/Phchr1/Phchr1.home.html) | not defined in the genome database |
| Phycomyces blakesleeanus | ESTEXT\_FGENESHPB\_PG.C\_10057 | a | F1 | DOE Joint Genome Institute (http://genome.jgi-psf.org/Phybl1/Phybl1.home.html) | exons 2+3 removed (GenScan prediction) – a splice site mispredicted just before the real protein terminus |
| Phycomyces blakesleeanus | ESTEXT\_FGENESHPB\_PG.C\_340109 | b | F1 | DOE Joint Genome Institute (http://genome.jgi-psf.org/Phybl1/Phybl1.home.html) |  |
| Phycomyces blakesleeanus | FGENESHPB\_PG.17\_\_92 | a | F2 | DOE Joint Genome Institute (http://genome.jgi-psf.org/Phybl1/Phybl1.home.html) |  |
| Phycomyces blakesleeanus | FGENESHPB\_PG.2\_\_465 | b | F2 | DOE Joint Genome Institute (http://genome.jgi-psf.org/Phybl1/Phybl1.home.html) | exons 2+3 manually removed (+ GenScan prediction) – a splice site mispredicted just before the real protein terminus |
| Phycomyces blakesleeanus | ESTEXT\_FGENESHPB\_PG.C\_670002 | c | F2 | DOE Joint Genome Institute (http://genome.jgi-psf.org/Phybl1/Phybl1.home.html) |  |
| Postia placenta | ESTEXT\_FGENESH3\_PG.C\_1110045 | a | F1 | DOE Joint Genome Institute (http://genomeportal.jgi-psf.org/Pospl1/Pospl1.home.html) |  |
| Postia placenta | ESTEXT\_FGENESH3\_PG.C\_810014 | b | F1 | DOE Joint Genome Institute (http://genomeportal.jgi-psf.org/Pospl1/Pospl1.home.html) | exon 2 manually extended to correct frame (splicing acceptor site changed), results in extending the N-terminus considerably up to the length of the other F1 paralog |
| Postia placenta | ESTEXT\_GENEWISE1.C\_160061 |  | F2 | DOE Joint Genome Institute (http://genomeportal.jgi-psf.org/Pospl1/Pospl1.home.html) |  |
| Rhizopus oryzae | RO3G\_06953 | a | F1 | Broad Institute (http://www.broadinstitute.org/annotation/genome/rhizopus\_oryzae/) |  |
| Rhizopus oryzae | RO3G\_08863 | b | F1 | Broad Institute (http://www.broadinstitute.org/annotation/genome/rhizopus\_oryzae/) | N-terminus manually extended, based on aa homology with RO3G\_13784 and P. blakesleeanus F1 |
| Rhizopus oryzae | RO3G\_13784 | c | F1 | Broad Institute (http://www.broadinstitute.org/annotation/genome/rhizopus\_oryzae/) |  |
| Rhizopus oryzae | RO3G\_06481 | a | F2 | Broad Institute (http://www.broadinstitute.org/annotation/genome/rhizopus\_oryzae/) |  |
| Rhizopus oryzae | RO3G\_07636 | b | F2 | Broad Institute (http://www.broadinstitute.org/annotation/genome/rhizopus\_oryzae/) | exon 1 manually extended (different splice site selected) to restore a conserved region |
| Rhizopus oryzae | RO3G\_11583 | c | F2 | Broad Institute (http://www.broadinstitute.org/annotation/genome/rhizopus\_oryzae/) |  |
| Rhizopus oryzae | RO3G\_14587 | d | F2 | Broad Institute (http://www.broadinstitute.org/annotation/genome/rhizopus\_oryzae/) | exon 3 manually extended (different splice site chosen) to restore a conserved region, also based on exon/intron pattern homology with other R. oryzae and P. chrysosporium |
| Schizosaccharomyces cryophilus | Scr\_CSL1 |  | F1 | Broad Institute (http://www.broadinstitute.org/annotation/genome/schizosaccharomyces\_group/) | GenScan prediction + exon 1 added manually (based on homology with S. octosporus F1 and the existence of introns in other Schizosaccharomyces F1); not defined in the genome database |
| Schizosaccharomyces japonicus | SJAG\_04092 |  | F1 | Broad Institute (http://www.broadinstitute.org/annotation/genome/schizosaccharomyces\_group/) |  |
| Schizosaccharomyces japonicus | SJAG\_03484 |  | F2 | Broad Institute (http://www.broadinstitute.org/annotation/genome/schizosaccharomyces\_group/) | N-terminus extended (GenScan prediction) |
| Schizosaccharomyces octosporus | SOCG\_03749 |  | F1 | Broad Institute (http://www.broadinstitute.org/annotation/genome/schizosaccharomyces\_group/) |  |
| Schizosaccharomyces octosporus | SOCG\_04002 |  | F2 | Broad Institute (http://www.broadinstitute.org/annotation/genome/schizosaccharomyces\_group/) |  |
| Schizosaccharomyces pombe | SPCC736.08 |  | F1 | GeneDB (http://www.genedb.org/genedb/pombe/) |  |
| Schizosaccharomyces pombe | SPCC1223.13 |  | F2 | GeneDB (http://www.genedb.org/genedb/pombe/) |  |
| Ustilago maydis | UM05862 |  | F1 | Broad Institute (http://www.broadinstitute.org/annotation/genome/ustilago\_maydis.2/) |  |
| Ustilago maydis | UM06280 |  | F2 | Broad Institute (http://www.broadinstitute.org/annotation/genome/ustilago\_maydis.2/) |  |
