# Supplementary material for: N-Termini of Fungal CSL Transcription Factors Are Disordered, Enriched in Regulatory Motifs and Inhibit DNA Binding in Fission Yeast
Source: PLoS One. 2011 Aug 12;6(8):e23650. doi: 10.1371/journal.pone.0023650 (PMC3155561; doi:10.1371/journal.pone.0023650)
Supplement: Table S2 — Cbf12 phosphopeptides identified by mass spectrometry (DOC). (DOC) [file pone.0023650.s002.doc]

**Supplementary Table 2 - Cbf12 phosphopeptides identified by mass spectrometry.**

| **Cbf12 phosphopeptide sequence^a^** | **TSC^b^** | **umod^c^** | **Mass** | **FDR^d^** | **# PO_4_** | **Site(s)** |
| --- | --- | --- | --- | --- | --- | --- |
| RPssEDIKTQEFYDSTR | 12 | 2 | 2217.97 | 0 | 2 | S10, S11 |
| RPssEDIKTQEFYDSTR | 18 | 2 | 2137.97 | 0.01 | 1 | S10/S11 |
| RVAtAIGSINANLEsPQLYSLAK | 2 | 26 | 2575.31 | 0 | 2 | T31, S42 |
| RVAtAIGSINANLESPQLYSLAK | 7 | 26 | 2495.31 | 0 | 1 | T31 |
| RVATAIGSINANLEsPQLYSLAK | 8 | 26 | 2495.31 | 0 | 1 | S42 |
| VAtAIGSINANLEsPQLYSLAK | 3 | 46 | 2419.21 | 0 | 2 | T31, S42 |
| VAtAIGSINANLESPQLYSLAK | 6 | 46 | 2339.21 | 0 | 1 | T31 |
| VATAIGSINANLEsPQLYSLAK | 66 | 46 | 2339.21 | 0 | 1 | S42 |
| STsLQEPVR | 5 | 7 | 1095.53 | 0 | 1 | S53 |
| IYGDsVsPAISSSK | 1 | 30 | 1569.7 | 0.03 | 2 | S64, S66 |
| IYGDsVsPAISSSK | 6 | 30 | 1489.7 | 0 | 1 | S64/S66 |
| IYGDSVsPAIsSSK | 1 | 30 | 1569.7 | 0.03 | 2 | S66, S70 |
| GDSVSPAISSSKAHstSSVsPY | 3 | 3 | 2310.01 | 0 | 2 | S76/T77/S78/S79/S81 |
| GDSVSPAISSSKAHstssVsPY | 2 | 3 | 2230.01 | 0 | 1 | S76/T77/S78/S79/S81 |
| GDSVSPAISSSKAHstssVsPYYSEKNESQAL | 4 | 3 | 3379.54 | 0 | 1 | S76/T77/S78/S79/S81 |
| AHstssVsPYYSEK | 4 | 15 | 1701.7 | 0.03 | 2 | S76/T77/S78/S79/S81 |
| AHstssVsPYYSEK | 3 | 15 | 1621.7 | 0 | 1 | S76/T77/S78/S79/S81 |
| KVNMNSNVPSSDSVRNssPNQYY | 33 | 11 | 2666.18 | 0.02 | 1 | S263/S264 |
| NSsPNQYYASTSK | 23 | 22 | 1525.64 | 0 | 1 | S264 |
| FPTPYsPSVPFGTYQEK | 7 | 75 | 2023.93 | 0.02 | 1 | S331 |
| SSVYFQQPLsR | 2 | 39 | 1390.66 | 0.03 | 1 | S378 |
| SLVNRPssAVCEPAR | 8 | 58 | 1664.8 | 0 | 1 | S433/S434 |
| NDsIPMMVYSQPVTIEQR | 3 | 52 | 2187.01 | 0 | 1 | S444 |
| NDSIPMMVYsQPVTIEQR | 2 | 52 | 2187.01 | 0 | 1 | S451 |
| NIFyyNADGALISPETDIAK | 2 | 57 | 2294.08 | 0 | 1 | Y534/Y535 |
| NIFYYNADGALIsPETDIAK | 5 | 57 | 2294.08 | 0 | 1 | S543 |
| QSSEISFTSGELEYsDPNDPTK | 4 | 56 | 2510.07 | 0 | 1 | S806 |

Rows without shading correspond to the N-terminus of Cbf12 and shaded rows denote peptides from the core region of Cbf12. No phosphopeptides were detected in the C-terminal region of Cbf12.

^a^ Lowercase letters indicate phosphorylated residues. Note that in some cases, the modification(s) could not be assigned unambiguously. In these cases, a conservative minimum number of detected phosphosites was considered.

^b^ Total spectral counts.

^c^ Total spectral counts for unmodified (non-phosphorylated) peptides.

^d^ False discovery rate.
