# Supplementary material for: N-Termini of Fungal CSL Transcription Factors Are Disordered, Enriched in Regulatory Motifs and Inhibit DNA Binding in Fission Yeast
Source: PLoS One. 2011 Aug 12;6(8):e23650. doi: 10.1371/journal.pone.0023650 (PMC3155561; doi:10.1371/journal.pone.0023650)
Supplement: Text S2 — New and corrected CSL cDNA sequences (RTF). (RTF) [file pone.0023650.s004.rtf]

Text S2 - New and corrected CSL cDNA sequences.
coding sequence in uppercase; introns in lowercase; yellow areas denote the corrections made

>CNAG_01173_new_cDNA(intron 2 included)
ATGTCCTATCAATGGAATCACAACCACGATACATCGACCTCCCATCGGGATCAAGACCCAGTTTACCAGGACTACGACAAACAGAATGTAGGCTATACTCCGATGCAAGGCCAAGGGATGCTTGATCTTATGGGCATCCCTCAGCCAATGTTCTTTCCAGGGATTCAGACAGAATTCAACAACGGGAGAGCCTCAGTCGGTGTAACAGGTGGCTACCAAAATAGTATGCCGCCACCTCAATACCAGTTTCGACCTGCTCTAGCTCTTGACACCACTGGCGGATACTTGGCCCCTCAGCCTCAACCACCCTCAGCATATTCCGAACTACCGTCTGGGCTCCGTACTGATTTGGGATCGAACACAGATGGCGACCTTGTCACGCCAAACAGTTATTCATCTGCTGGCGTCCAATACCCCCCTTATAACAGCGACTTCATGACATCGCTGTCGGTAAACACGGGTATGGTCAACCCTGGCCAATCCGGTCGAGGCATTGAGCAACCCCAGCCAATGTTGGGAACGTATCAACCCACGGTTATGCCCCAGTATGGCTACTACTCCATCCCACATCCTGAAATGCAACAGGCTCAGCAACCTGTTGAACCGTCAGTTCAGCTGGGAACGATCTCTCCGTCAGAGCTTGGACAACGTCAACCTTTGAAGCCTACAAAGAGTTTCAGTGACCTGATGATGGGCAGTCGTGCATCATCATCGTCGTCCTTAGCCTCTCAAGACGTACCGGATGGATGGAATGGCAATGCTCTCGAAGATTGGACCCGACCCCTAAGTCGTGCATTGCCATCTTCAGGCACTCCCGTTAGCCAGTCTGCAGGTAGCAGACAACTACCTGCTGCTGCCGATGTATCTGCTCTTCCCGATCTTACCAAACGCCCGTCTTTCGCTTCCTCTCCACTTCGGAATCCTTCTTTCTCACCCCTGTCTCCTAGCGCGTCAGATACCGACTCCATCATCAAACAATATATTCGGGCTCCTAATCGCTTGGCTTTTGGAGAGCGTAAAATCATCGTGATGAGCCCGAAAGtaggccaaaagagttatggtacagagaaacgattccttgtacctcatccgcaggCAATTCTCATTGGTAGTGGGTGGTGGGCCAAGAGCCCTGACGGGTGTCCCGTCTCTCCTCTTCAACCTCCTAGAATCAACATCAGTTTGACAGGGGAGCAGGCGGTGAAGGACTCTATTATCAGCTGGACGGACCTGAACGGCAAGAACATGGATGAAAAAGCCAGCACTCAGGGAGTCAAGATTGATGACCAACCTTTTACTGGAAGTGTAGCAGGAAAAAACCTCCATATCAGCGATAATGACCCTAAGAGGAAAGAGGTCAAAGCATTGGTAACGGTTAAGGCGCCTCTAAATAAACATGCAGGGCCTAATGGATGGGGAAATGCCAAAGGGACTCTTTCAGATGTGTCAAACGACATTGTGTTTGGAATCTTTGAGAGCAAAGACATCAAAATCATCAGCAAACCGAGTAAAAAACGATCCACGGCCAAGAGCGGAGAATTAACCATCAGTCACGGCACTACCGTTGCTCTTTTCAACCGTATCAAATCGCAGACGACGTCAACTCGTTATCTCTCCGTCGTACCGGATTTCACACGTACTCTTGGCTCTGATGGCCGGCCAGTCACCGGTGCCAAGACCCCTCAATATGCAGATGAGAAAGGCGCTTTGAAGGGATTTACAGCGGATGCTAGCTTCTGGGAGTCTTTCATCATCTGGCTTGTCGACCCTTCCCTTCCGTCTGGTCCAAGCAATCATCAGCCCCCTAATCCCGATTGGCCTCGCCCCCCTGCAAACATCATCCCTCTCAACACGATCTCTCATTCTATCCGCTATAACTCGACTGTCGTCCTTCAATCACTTCGAACGGGCGTTATCTCTCCTACCCTTGTGGTTCGAAGGATTGAGACTGATTCAGATGCTGTGGGGATGGACGGACATGTCCATGAAGTACCTGCTATGCCACCCGGTGAACTCGCCAGCGACTTGGTCTCACAACTACAAAAAGTCGCATTCGAGATCTACAGTCCCGATACCATGAATCGCCTTCAACGGGAGAGTAAGTATGGGGGATCCTGGTTATCGTGTTACCAAGACGAGGTGCGGGAACATGCCATCAAGGCTGAAAGGAAATGGGCCGTCCTGCAAGCTCCTGCGGGTAGTAAGCCGGCTTCCAGACCGAACAGCCTACCGAATACACCTCAACAAAGATTCGGTATTTTGCCCATGACGCCCCACACCAATAACATGAATCTTCCTTCTACCCCGTCCAGCCCAGTCAGCTCTTCGTCCTCCCTGGATTACTTTAACTATCAATCTCAAAGATCGTCCGGTCATAACCACCCTCTGATGTCCCCAGGTAGCGTTGAGACCGCCCTTCCATCAACAGATGGCGGGCCTATCAGACGGCAAAGAACATCATCCATGGGTAAAGGGCCTTTGTCGAGACCATTACATAGGAAAAGGTTGTCTACAGATTCAACAGGATCGAACTCTTATGAGTACATGCCGAGTCTTTCATCGGCCATGTCAAGTGCAGAACAGCCAGTGCAGCCTCAAAGGATGTTTTGGACCATGGATGTTGGTGACAGCTGTGTTTGGAGTATAATCAGCGTTGAACAAGCATCATACACGTTCTTCACGCCTCCTATTTCAAACTTTGAGGAATTCGAACCTGTGGCGCCTTTCCCTGTGGCTAATCGCCTTTTGCCTGCAAATCTCAGCGCTGAAGTGCCTTCCAAGTACGCGCATCATTACACGACAACAACCAACATGCCCCTTGTCACTTTATATGGGAAACATTTTGTCAAGAATGTGGATGGGCTTGCGAAACATGTAATGTACTACGGAGACGAACCGGCGCGGCATAATGAAGTCAGATGCGCTGAAGTTATGGTGGCATCTGAACCTGAACAATCAGCTAAGTCAGACAGGAAAAGGCCTATATTTCTTGTTCGAGAGGACGGGAAAGTTATCATTCCGACTCCACTTCATTACCCCGTATAG

>SJAG_03484_new_cDNA(N-terminus extended)
ATGTCGGGCTTAAAAAATCATTCTCCGTACGGAATTGCTCCAAATACAGTAAATTCAGAAACGCAAAATGTTGCCCGGCCGCAAGATGAAAATTCAAACTGGAACATGGAAGTCGCTTATGACCGACTGGAAACAAGAAGCCCCTTAATTGCGCGGTTGAATACTGTGTGTCCTCCTACATATATGAACCCGCAGGCACCTTGTTCTACATACGACGATTCCAATATTACTGAGCAGCAACGCTACTCCTCAGGCTTTGTCGCTAGCAGTGCCGACCCCTCACTCTATCCCTCACAAATCCCTCGCGTGGGTTACTACGTGGATAGTGCCGATCCCTCCCCTTATGCAGACGACACTGGAATTCGAACGCCTGGCAATGAAGCCAAGCTTGGGATGGCTTCAGAGTTGCCTCAAATGGGTATGAACAATGATGCTAATTCCGTGCTACGACAACAACAACAACAACAACAGTCGCATCAACAGCAGCTACAGGCACTAACAAACCAATCTGCCTTTAACCATATGGGTTGGGACGTAGCTGGTCACTTTCTGCCTGGTGACTCTGGTCGTTTTCCCGCCGTAACAGACGCATTTTCGAGTCAATTCGTTCCCTTGCAACATCCCCAACACGATACTAACCCTTACCACGGACTCCCTAATGCAGGTGCCTATCCTGAGGTTTATCCGGCCGACCCGACCTCGTCGTATTCCGGCCCATTCACTGATCAGCAAGGCAATTTCGGCTCATCGTTCGGTGACGTCGTGCCCGGCGGAAACCCAACATCTTCGAACTCACCAGCAGTGCTAGAGCTGTCAGGACACTCACCGACCCATCTCTACGGTGCTGGTGATGCTCCTCGTCTGCAACAACTTCACCAGCAACCGAGACAGCAGTTCCATGTTTCTGACTACGGCCAGTTTCACGTCCCAGCTGGAACCAACAACCCAAATGCTAATAATGGCTTTCGAGTTCCGTTTGGTGGGTCCAGCCCCGGCCCAGAGTTGAGTCAAACATCTTTCTATGACGGGCCAGAGTTTCCAGCACCCGCTGAATCTTCACATGCTTCCCTTTTGAGGCCCTCTACGTCTCACTCAGACTCCATGGGCTTAAACGCCCCCGACAGAAACAATCTTTTTCAACGGGTTAAAAATGTTCTGCGACACCCAGAGCTGTTGTGCGCTATGAAAGTATTCATGCCCTCCCTGGGTCAGAAGTCGTACGGTAAGGAGCGTCGCTATATATGCCCGCCTGCGGTCGTCTACTTGCTCGGCAGCAGCTGGTTTCGATGCCCACTCGACAAAATAAATATCATTGCGAACGCGGCTGATGACCCTGACAACCTTAAAACGTCAGAGACGCCAACCTTTTACACGAGCTCTTCCGACAGCGCCACAAACCTTCTTTCGCTGGGTCAGGTGAAACTCGACGATCCATTGGAGCAACAACAATCACCATCTCCCATATGGGCGAATACGGTGTTGAAAACGCTTTATTACTCCGGTAAGGGGGATCACAATACGTATGGCCGCTCAACTACCCTGCAGGTGCATGTTCGGACCCCACAAAAGCGGATAACTATGGATAAGCTTCGTATTGGCATCATATCGAAGCCGAGCCAAAAAAAAATGATGATGAAAGTTTCGGATTTGAATATCTGTCACGGCGATTGTGTGAGTTTATTCAATCGGTTTCGCTCTCACAACAACCTCCCACGGTATTTGTGCACAAATGTCCTTAATGACGTCGTTACAAAGCGAACAGAACACTTACAATTCCATGAGGAGTTTACGCCGGCCACAGACTTAGACTTAATGGAAGCGTCAACATGTCGATTAATTACAACAAACACCGTTTGGGAGCCATTCAATATATATTCTGTGGAAGAGCTTGAAAACAAATCAAGTTATGATCGGCGTAACACAGTTATTTGCTCCAATATGCGCATTATAATCCAAAGTCAAATTACGGGTGTGCGTTCACCTCCGCTCATTATTCGGAAGTACGAGAACCGTAAAGCACTTGTGGTGGAGGATGACCAGTTGGGCGACTCGATCAATTGTTTGTCTCGATTAGCGTTCCAGTGTCCTCGTACAAAGTTATTTTTGAATTTGGACGAATTTAGCAACGGTGAGATCCGATTTCTATCGGCTGACCCGGCTCCTGGAGAGGGTGATGGAAATGGGGAATATGTGAATCTTCCGTGGTCTGCAGTGTGGTCTATCATTACCACTCAGTCCGTTCGAACAATGTTTTTTGACGACTGTTCCGGCAATGATGGCATGCTCTCCATCCCCTCAGCACCAATCATCAAGTTCATTCGCATGGATGAGAGCAATATGTTGCATGTGTATGGCGTGAACTTCACCGCCGACGCCCAAGTCTGGATTGGTGAAAATCCATGTCAGACGTATTCGGTGACAGATGTCGAAATCGACGAGCCCACACTGCTTCGTGGACTTATTTCTACTTCACACGTACCTCCGCGACTGCATGCATATCTTGCAGATTTGGCTGATATCATTTGTCAGCCGCCGGCGCTAAGCGTAACTACATCTCCAGAGTTACCGATACTCGTTTCGCAACAAAACATCATTTTCCACAGTGGTTTCACCTGGCCAATCCATCCACATACCCTGTGA

>ScrCSL1_new_cDNA(brand new prediction)
ATGGCAGGTTTTGGCAGCTGGAATATACCTGAATCCAATGAAAATGACTCTCTCACATTGGATAATCTAAAAATTGAAAACTTAAAAAATGAACTAATCCTTCGCGGAGATTTGAATTTGGATCAAAATAATGACCAAAACGTAAATACTGAGGCGATGCCAATTCAAGTCCTTTCTTTATTTAGCGCTGTTAATTCAGCGCTTACTAATGTGGAAAAGACAGAAACAGGTTCTCGTCATGTACAAGCAAAACTCGAAGATGACCCAAAGGGTCACTTTTTTAACAGCCTTTCACATATTCAGATGAATGAGCAAAGCAGAAAACGAAAAAATATGGAAGATGAAAATGTTTCAGAAAACTCTATAAACGACCTCATTAGCAAAAATATCGAGTTGCTCTGCCAAAACAACCTAAATCAAGGAGCTGACCTTTGGTTGAATGATGCTAGCAAGGGAATGAAAGATGGAAGAAGCCAAATGATAAAAATCTCATGTAGACATGCCTCCGTCATCCAAAAATCGTATGGTTCTGAAAAGAGATTTCTTTGCCCTCCTCCCGTCCTCATGATAGATGGTCCATTTCAATCTTTGCTTGGCTTATCCTTTCGAGTTGAGATCTCTATTATGAATGAAGAAGGCCAGTATTCACAGGCAATCAGTGAAATATACAATAATCAACAGTGTATGGTTTTTCGATCTTTGCATGTATCTAGTCTAGCAGCTGCAAAGTCGAAAATCTTAAGGCTTTCCATCGATTTTGTTTCGACAGTTACGGACCAAAGAGTATCGCATTTGGTGACTTCCCCTATTGACGTTGTATCAAAGCCGGCCAAAAAAGGCACAAAATCTAAGGTGAGTCATATAACACTGCGTTCCGGGTCCGTCATAAGTCTTTACAACCGAATAAATTCGCAAACGGTTCGCACAAAATATACAACATTAGAAAATCGGCAATTTTGCCTAAGGAGTGATGGCTGGGCACCCTTACGTATTCATCTAATTTCTTCATCACCCGAGAGCACCACAGAAGGGATCTTGAATAATTCTGTAGAAGCGGTTCCAATTAGGTATGGTTCTGTTATCCAACTACAAGATGAATTTTCAGAAATTATGAGTGACCCTTTAATTATCCGCCGAGTAGAAAAAGACGGGATTGCTCAAGATGATGGATACGTGAATCAAATGCACCGCATAGTTTTAGAAAGCTCAGCTTCATTTTCAACAAATTTAGCACTTGGATATGAAAACGCCAGAAGTGGGTTTATGCCGGCTGATCGCACACAACCTCGATGGTTTTTGGGTGCAACTTCCGCTCAATGTAAGAACTTACCTAATGAAGCAGTTGTACCAGTTGAATGGGAACCAATAGAGACTTATAATGATGAAGTATTAAAAATCCGTGATTCTGTTTGTTGGACTATCGTTGGAATTTCCCAATGTGATTGTACAATTCTAAGACCAGTACAAAGTCAGTTTGCTCTTCGACATTTAGATTTACCATGCGTGGAAGCCCCGCCTATTTATTTGCCAAGCAATCAGTCATTAGAGTTAAGTATAGAAGGGTTTTCTCCGGACATACAGATATGGCTAGGCAATATAGGGCCCTTGAATTACACGAAAATAGAGAAAGCAGAAAAAAGCTTATCTAGGAGTGTCACAATTCTGGTTAGTTTGATCCAGCTTTCTACGGAAGCTATCACCTGCGAAACACTTCCCCTGTTATTTGTTTTTCCAGACGGCACCATTGTATCGGGACGATGCGACATAAGCCTGTCTCCATGGCTTTCCCTTGGGAAGTGA

>Lb_EU2.LBSCF0005G03540_new_cDNA(N-terminus truncated)
ATGTCGCTCGATTTTCCTTCTGGCAACGGACAACCCCCGCACAGCCTCTACAGCCAACCAGACCTAGATCAGCCGTTTAATATGCACAACTCTGATAGTAGTTCCACGTCGCCCTCTCATCCCCATATTGGTGGAAGTCTACAGCAGCACACCTATGATCACCCAGACAGGATGGAGGCTACCGACACTACCAATTATGACCTTTTCCCAAATTCTTCGTCCGGTGCTTCTTTTTCCTCTAACCGATACCGCACGAACGCGTCATCTTCTTCTTCTCTTGGTCACAATTATGGGATGCACTCAGAGGGAGTGTATTCACATTCCTCTTTCAGTGATTCTGTACCTTCATTCAACGGCTCCAACACCAACCCATACGACATGATCAGCAGCTTGCCTTCTTCATATAGCAGTGACAAGATTTCGCCATTAACTCCGAGCGACTCAGTAAGTGGTCTTCACCATTCCCCTGCTTATCCCTCAGGAAAGGATTATCCTACACCTGGTTACGGAGATATGTCTGAACGTCGCCTTCCCAACGGAAGTTCCACCGGTTATCAGTCCGAATACTCTGACGAGTACGGGATTGGAGGTGTGAACAACGGGCTTTCCTTCCCACCCTCGACAATCCAACATTTCCAAGATCGTCTGGGTCGTTTTCCACCTGATCGCTACAACCACGCCTCAGGCCCTACGATTCCCTCACATCATGGCCCTGACATCTTGCGAAGTGTTGCCCCACACGCGACTCACTCTTTCCGAGAAGGTGGTGTTGCAGGCTACGACGAGATGCCGCATTATATTGGTGCCAGTCCTCATCAGGATGTGGCCCTCCGCATGCCTTCTGTCGACGAAACCCTGGCACGCATGAAGCTTCAAGGGCATCCCATAATGGGCACTTCAAATGATCTCCACACATTCATTCGTCCCTTTCTTGATCAGTATGTTCGCACGCCCAACCGACTAGCCTTTGGAGAGCGGACCATTATTGTCATGTCGAGCAAGGTGGCCCAAAAATCCTATGGTACGGAGAAAAGATTTCTATGCCCACCACCAACCGCCATAATGATTGGCAATTCATGGTGGACTGACGTTGTCCGAAGGGGTGAAGACCCCAAACTATACCCTCCTCGAGTGGTCATTTCTATCTCTGGCGAGCCCGTGCCGCAGGAAGGATCCATTGAGTGGACCGGATCATCCGGTAAGTCTTTCGATGTGGGCGACCCTCCCACAGGAACAACGTATATTGGGCGCTGTGTTGGGAAACAACTTTTCATCTCCGACGTGGACGAAAAAAAGAAGAAGGTTGAGGCCCTGGTCAAAATTACAGCCCCAGGTTCGGAGGAAGACTCTGAACGGGTGATTGGGACGTTCCCCAGTCGGCCTATAAAAGTCATTAGTAAACCTAGCAAAAAGAGGCAGAGTGCCAAAAACCTCGAGCTCTGCATCAATCATGGTTCTACTATTTCATTGTTTCACCGTTTGCGTTCTCAAACGGTCTCTACAAAATACCTTTGTGTCTCTGGCTCTGGATCTTCTTTCAAAGGCTCAGATGGCGCCCCTTTAATGGGACTTGACCAACGGTCCCGGTCGACAACACCATCGTTCATTGCCCGAACGGCCTCCTGGGATCCTTTCGTTATGTACATCGTTGATGTCAGCAAACCAGCTGGTGGGGGAGTTGACGCTCCTCCACCACCGCCTCCCCAACCCGATTATCCTTCCCCGCCCCCAAACGCCATTCCATTCACAAACAACGGGTCACAAATCCCCATCTACTATAACCAGACTGTGGTTTTGCAGTGTCTGACTTCCGGTGTTGTTAGTCCCGTATTGATTATTCGAAAAGTTGATCGCCAGACAACGGTTGTTGGTGGTGGACTCCAAGAAGGTGCCAAGGGTATCGCAGATCACTTCTGTTCTCCTGGAGAAGTGTGCGGAGATCCCGTATCTCAACTTCACAAAATCGCCTTTGAAGTCTACGACTCCACAAAAGGATTGCCTGAACCTGGGACGCCTGGCGCCACAGGCGCTTTCTTGTCATGTATGGGTGAGAAGGTCAATACTTATCGCCCAATCGATGGCCGACAGTGGAACAACAATCAGAATGGATCAAATTCTCCTGCTCTCCCTGGTTCGCCCATCTCTCCTACCCCTGGCTCAGCCAATAGTGAATATTTCGGGCTTGGGGATAGCGCGCCCGCGTCACCTTCTGCCACGGACTTTATGTCTAACGATGGTGGTCGTGTTAAGAAAAAGAGATCGACTTCAAGTGCTGGTGGTATTACGAAGAATGTAGGAGCTAAAGGCCGCCGCCGACCGAGCTCAGCAGGCTCCGTCTCTTCAAGCCGCCGTGGCTCTGGCAGCGACGCCGGTGCCTCTTCAGGAGCGCTCTGGCAAGTAGACATAGGCGAGACAAGCGTCTGGACAATTGTGGGAACAGACCAAATCCGTTACAATTTCTACGTCCCTCCTCTGCTTTTTGATAATCAGCATGCGCCTCAAACGGGCTCATTCCCTATACCATCCAAACCTATTACCCCATTCCCCGGTGTCGTCAAATACCTTCCCCCTGATCGCGCCGCCGAGGTTCCAAAGTCCAATTGTGTGCAGTCACGCGCTATGTTGTCAAAACCCAGTCCTCACGCTTCAAAAATGCTCACTGTATACGGGGAAAACTTTTCTAAAACCGACCCCGTGAATGTTTTCTTCGGCTCTGAGCCTTCGCCATACGTTGAAGTCCGCTGCACGGAAGTTTTAGGCTGTTTACCCCCGGAATCAGCGCCCTCTAAGCGTCGTCCCATTATTCTTATCCGTTCTGACGGTGTTGTTTTCCCATCTAATACCATGTATCCTTAG

Pp_estExt_fgenesh3_pg.C_810014_new_cDNA(splicing pattern adjusted in the N-terminus = exon 2 extended)
ATGCGAACTATCCTGCCGTTTGCTTCGGATCCCCTTCACCTCCAGATGGCCTCGGTCACCACTGCGGGTCCTCAGGACCACCTCGTCCCCCCAATTCATCAGGATTTATCGCGTTCGGCTACCCCACAGCAGCCAGACCTGAGCTTGCTTgtctcgaatcatcatgcatctctaacaaacggccaacatgcagactcaccgtccacagACCAAACTATCCAGTCCATACTAGAGGTAGCCAAGCACGTATCCGCTCCCAGTAACGGACCACCAATAACATGGAACAATCTCCCGCCACCGCCGCCACCGGAGAATAGCAGCGGACCTGGCCCGTCAACGCAATCGCAGCGAGCAAACGTGAAACGCAGGCTGGAGGAATCCGACGTAGAGAACCCTCACAACAAAATTCGACGCGTCGTACAGGACCATGTCTCGCATGATCCTGCTCGGGTGATGCCCATGACGACCGTGATTTGTCTGCACGCTGCGGTTGCTCAGAAGTCTTATGGCACCGAGAAGCGTTTCCTTTGTCCTCCCCCCGTGGTCCACATCGAAGGGCCAGTCTGGAATATGAAGAATCAGCAACTCGCTATGGCTGTCCTTACCGAGGCAGGGGAGCGCTCATTTGAGCAGAAAGCGCCGCTAGACCAAAGTCTCACCGCCAGCTTCAAATTCCTGCACGTCTCGGGGACAGCCAAGACAAAGAGTTTCCAGCTTTCACTTGACATCGCGGAGCCGCCCGCAACTAGCGCTGATGGCTCAGAGTCGGATAATGGGCGCATCTGGGCGTCGTTCGACTCCGCGCCCGTAACGATCATCTCAAAGCCCTCGAAGAAGACGGCTAAGACGCGTAGCGTCTCTTCATGCATCCTCGCTGGAGGCCCCGTCTCGTTATTTAATCGCATCAACTCACAGACTGTACGGACCAAATATATGACTGTTGACAGCGGACAATTGTGCGCGAGCAACGTTGCATGGTCTGCATTCAATGTAAACGTAGTGAGCCGCCCTCCAAATGCAAGCAATGATCCTGGTCCCCTCGCTGGTCCTGGACCGCAGACGGTGTTATATGGCAGCAAGGTTATCCTTTCCGACACGGTCACCGGGACCCCAACCCCGCCCTTATTCATACGGAAAGTCGATAAGGGTAGAATTGCTACTGATGATGGCGGTCCCGTCAGTCAGATGCAGAAGATTGCGTTGCAACGGGTGAACGCTGACGGTACTCGACATTACCTTTCCGCAGCAGGGCCCATGTCCGGTCAGCCTGGTGTACCTGTGCCGCCTACACCGGGCCTTGCGAATCAAACGGGAACGCATCCACTCTTATTCTCGACACCTCGGGTTCGCGAAGAGGTCAAAGACAATCAGAACGTGATAATCGACGAAGTCGATGACTATCTGTGCTGGACGATTGTTGGAATATCTAAGTTCCAGTATACTTTCTTCGATGCGTTTGGCCAGAACAGCAAGATCCCTGATCTCCCGATCACACCGTTCCCGACCCTCTTCACTGCGCCCGTGCATCGATTTGAGCGCGAGCGCGGCACAGAACAATTGGAGCTGACTGCCTCCAATTTCTTCTATGAAAATCCAGCGACGGGAACGGTCGAGCCACTTGATGTTTATCTCGGCCCCATTGGCCCTTTGCATATCCGCATCTTCCACTCGTCGGTCCCAGTGGGCCAGGTACCAATGCCTTTCATACATCCTCCAATGCCTGGCGACGCCCCTATACCTGTGGACCCCAGCACTTCGTCATATCCCACGGGCAGACGGCCACCGGGTGCCCTTCATGGCCCACTTACCACGACTGTGCTTGTTGACTTGCCTCACAAGTCGGACGTCATGAAGGCGCTCGAGGAGGACGCGGTCCCGCCGGCTGGCGAGAGCGGAGACGTCGCTGCGAATAGGACGCAGTCGAAAGTGGAGAGGCTTTCGGGGCGGAGCCTTCCTCTGCTATTCATTCGAGCCTTTGACGGAGTTGGATATCACTCTGGGCGCGCGGTAGCCTGCGAGTACATATTCAAGGCTGTCGATCTCCTCACTCCGCCACCGGGCGAAGATGAGAGAGAATGGGTTGCGGAACAAGCGGCGCAGGCCGCGGAGAACAATCTAAACAGCTGGGCTTTCAGGGTGCTATGA


RO3G_08863_new_cDNA(N-terminus extended)
atgaatgaagaagaaccagtagaatcacatataagatcagaacaaatatttggtagcagtagaaagatccaattcattggtctatcgaaaccaccccttccaccacaacaacaacaaaaggatcaaATGTCGATTGAATCCTTATTGACCTTTTCTCATCCACGTCAGTCACAGACAGCTTCTACAAATGATATCACCATCAATAACCTATTGACTTGCGACATGAAACGAAAATGGGCTGAACCCAGCTTATCAGTAAGAAAAAAGTTGAAAAAGAAAAGCAAGGTGGTTTGCTATTATGGTGCCGTCGCACAAAAGTCGTACGGTTCAGAAAAGCGCTTCTTGTGTCCACCACCGATCGTTCAACTGAAAGGTGCGAATAAAGAGATGGAACTGTCGATGTCGATTTTATGTGAAGACAATGACATTCTAGAACAGCATACAGCCTTGGATGAGCATCACTCTGGCAGTTTTCGTTATCTGCATGTCACAGGCACAGCCAAAGCCAAACAATTTAAACTTCAACTCAACCTATTGCAAAATGAGTCATCCTTTGCTTCCTTTCTCTCTAAGCCCATTTCCATCATTTCCAAACCTTCCAAAAAGACGGCCAAGACCTCCAGAAATACCTCGACTTGTATTCTGGCCAATTCACCCGTCTCGCTCTTCAATCGGATCAATTCACAAACAGTGCGTACGAAATACATGACGTGCGATCACAATCGGTTGTGTGCCAAAAACACGACATGGTCACCGTTTGATGTCATCGTGCTTTGTCAACCGAAGCAGGTGGTCATCACCAAACAATCCAAGAGTTCACCACGCTACAATACCTCTCGACTGCAACTGCCCAAGACACCCGTCTCTTATCTGACCTACGGCACCGAGATCATCTTGCGCGAATCCCGAACGGGACTTTCCTCGCCCCCTCTGATTCTCTGTAAGGTCGATCGAGGCCGGTTGGTCGAGTCAGCCTATGGGCCAGTGAGTCAGATGCAAAAGATTGCGTTACGCTTAGCCTCTTCTGATCCACAACATCCTGTTTATTTAAGCGCGGCTGGTTCGATGTCTTCCGAACAGGATACCCATCAGACTTGGTTGGATTATTCTCCTTCAAGGAAGCTGGAACAAGAAGAAATTGATGATTACCTTTGCTGGACAATTGTGGGTATTCATACATTGGAAGGTGAATTTGATGAAAGGCAACCTACCACCCCCTCTTCACCCCCGCCAGAACAGCCCCGTCTTGTGAATCCCTATCCTTACCTGACCCATCTCCAGTACAAACCAGAGACGAATACGTTGGAGGCCATCGGGCAACATCTCATTCAAGCCGCACCTGTTCCTCGTCTGCTCGAGCCCTGGTTAGGCACACACGGTCCACTGCCGACCCGGATCGCGAGTCCGCCTGAGACGCACACCCCTCATGAGATACACTGGTCGATTGATTTGTCTTCTGTAAAACAACAGACGGAGGAACTACCCTTGCTACTCGTCCGTCAAGATGGTCTTGTGTATCATACAGGCAAATCGCTCAAGTGGGATCAAGAAGGAGAGGGTTGGTTATGTAAATAA

RO3G_11583_new_cDNA(2 5�f exons added, possible frameshift corrected by removing A1011)
atgtttacaactgaaacaagaaaaagaaaacaagatgaaatgaatacaactttgccaaccaattggacagattttatttattctacaccttcatcaccttcgatagataacttatttgatcaacattcttattcatttgattcaagcagtggaacaaacagtcgtcgtcattctgttgctgttggtgaattagattatcattcgtttgatttaaatagtttattagaagaaagacctttacataaacgtgcaatgtcattaagagaagatgatttgactgccaatttgttttcttcttatctatttgatttggttgatacaagaccaagagagttatcgatggatagcagtattatatctgacctttctttgaatgatttatcaaataataatcctgacttgtacaagtttaatacttctttggaaactattactccttctgctactctgaccaacgaaatcaattcaatggctgattggttattagaaaatcctcaaaagagaccaagacgttctactgattcaccccttggttcttcttctgattcttcttcttcaccacctattacccccatgcagcaggtctccttgggatttgaacctattcaagaagaatgggatcttcaacccttaattcaaaattatttacttcaaaaacaatcaagagaagactatattcctggtgaaagaacaatcatgattcttactagtaaagtagctcaaaagagctatggtaccgagaaaagatttttatgccctccgcctgcaaccatcatgaaaggtaccaattggtggacctcggacaaattgaccgataagaagacgccttctttatttcattctccgtcaaacgctcttcaatcacctaaactcacgatacatatctcgggtgaaacgattcagcagacgggtgtgatcgagtggcagacatcttctggtaacattatcgataacaatgcacaaaaggtattcggaagatgtatttcaaagcaactttacattaatgacgcagatgaaaagagaaagcgtgtggaggtcttggcaaagatccaattaggcaatggttccaatttgggtactttttcaagtaagggtatcaaggtgatcagtaaaccttcaaaaaagagacagagtgcaaagaatatggaattatgtattcatcatggtacaaccatctctttattcaatcgaattcggtcacaaaccgtatcgactaaatatcttggtgtatcaaccaccacacctcaacccgattcaaatggtacttgctttgtctctcgaaccggtgtttgggatccattcgtcatctggatcgttgatacctcttgttcacccaacactgccaatcgacctaaacataatccactcaatcctaattatcctcctccacctgccatcgccttacagacctcttctaccctagccattcattacaatcaacccgtcgtccttcagtgtgtcaccactggtctggtgagccctgtgATGATCATCCGTAAAGTCGACAAACAGAGCTTGGTCTTGGGTGGTAATCGTGTGGATAATCCGATCGGATCCCTGGGTGGTGAATGCAGTGATGAAACCTTGGGTGATCCTGTCTCTCAATTACACAAGGTGGCCTTTCAAATCGTTCAGGATCCTTCATTTCATCAAGGCAACCTGAAACAAAACACAGCTGGCCACTGGAAGATTCCTCAATCAAGTCATCCAGTGACCTATTTGGCCTGTCTGAATGATGTCGTCGGCATGCACAAGACAACCTCCACCCGTCACCTTGTCCCTCAGTGTCAAGAGAACACCTTTGGCGAAATCGCACAGGATCCTATTGTGCGTCGACTGAGTACGGGTGAAATCAAACGTCGGGGCAGTTTAGGAAAGGGATCGTCTGCACTCGACCCTACTGGGCTTGAAGGTGCTTGCTGGACTGAAGATGTTTCAGATGCAGCTGTCTGGACGATTGTTGGCACGGATTGTGCTAGTTATACTTTCTGGACACCGGATGAACGTACGATGCCCACCGCACCTTTCCCTGTTCTTCATGAACTCACCAAGAAAGGCAAGGATCGCTTGACACTGACCGGCGAGAATTTGAGTCCGGATATCGAAGTTTGGTTTGGTGATGTGAAATCGACCGAGACAGAATTTGTATCTCAAGATTCGGTACACTGTAAAATTCCTTTTGATGTTGCAAATAGTACAACAATTGAACAAGAAAATGATCACAGAAGGATTCCTTTACTCTTGGTTCGTGGTAAAGGAATTGTTTACAAAACGAACCTTTATTATATTTTATGA

RO3G_14587_new_cDNA(exon 3 extended)
ATGACTGGTATCCCTCCTCAACATGATATCATAAAGCACAGTATCATATTTCACAATAATGACACTAAGCCTCAACCTCCTTCTCCTGAAACACCCACAAGCAGCAGCAGCAGTCGAAAACGTAAACAAGATTTTCAATTCACTCCTGATCACCTTTCCTTTCCTCAATACAATTATGCCGAACCTGAAACACCTCTGGCCTTACAACACGAAAAATTCATTCAATCTTTACATCCAGATGGAAGCGTGGGAGAAAATGAAGAACTGATGGTCGTCAACTTTGATCAATCCAATCCATTCCCAAGGCTAACTCAACCGATTGACCTGGATGACCTCCTTCAGCAAAGACAAGCCTTTCAAACCTGGGACGCTTCTTCTTCATCTCCTATTCAATCTCCTACTCGTTATTCCCCAGGAACACCAGGTTTTTTCACGCCAGGATTTTTAGAGTCGCTTCAAGAACATCCAGTCTATGACCATTCGCTTTCGATCGATTATGGTTCACATCATTTCAATCAGGAATACAATCCACTTTTAGTCAAGCTGGAAGAACAGTCACCCGAAAAGAATTTGGTATCACAAAGTGGAGAAAGTGTCACGTCTTTATTCCCTTCTGATCCTGCTTCCATAGTAAGACCCAATCAAGCGCACGACAGCCCCATCCGTCGATCCTCCAGTCACACGACGGCCAGCAGCCCACACCGTCTTGTGCATCTCTCGCCACTCAAGATCAAACCCTTCATTCAGACTTACCTTGCACACGCCATCACCCAACCCGCCGCGACTCAGCTGGGTGAGAAGACGGTGATCGTGTTGACGAGTAAAGTGGCCCAAAAGAGTTATGGCACAGAGAAAAGgttcctctgtcctcccccgacagccatcttggtcggcacgagctggtggacgacgaaagaaaagatccaagacaaggaggaaacgttacgcatccccagCCTGGAGAAGGACATTCTCTTGGCGCCTCCTAAACTGACCGTGTCCATATCAGGTGAGACGTCGACGCAAGCGGGACAGTTGGAATGGTACACGGTCTCTGGTGCCACCGTCGGACAGACCGGACAGATCAAGCCTCCGATCAAGCCAGAATCCACCAGCCGTTTCCGCAGTTCAGAGTCTCGTCATCCTCCGGCGGACGCCTACAGCAACGAACGTCAGGAACTCTTGGCCGCCGGAAAATCCGTCTCGAAGCATCTCTATATCCATGACGCGGACGAGAAGCGAAAACGCGTCGAGTGCCTGGTGAAGCTTCAGTTGGCGAATGGACTTCAGCTCGGTCCACTGGCCAGTAAGGCGATCAAGGTGATCAGTAAACCGAGCAAGAAGCGACAGAGTATAAAGAACATGGAACTCTGTATCCATCATGGCACGACCGTGTCCTTATTCAACCGGATCCGTTCCCAGACCGTGTCGACTAAATACCTCGGTGTCTCGACCAGCAAAGGCAGCCCGCTGGCTTTCCCCGGCCTGGCCTTTCAACACGAAAAGAACCGGACGAGCGAAGGGACCTGTTTTGTGGCGCGAACGACCAGCTGGGACCCGTTTGTGATCTGGATCGTCGATACGTCAGCCAGCAGCGAAGAAGAAGGCGAGACGCCAGAGGATTATATCGGCCATCATGTGTTTGCCCGAAGCACGCCCTACCCACCCCCGCCTCCGATCGCGCTCAAGAACAAGACGGGCGGGCCGGTGCCGATTCACTATAACCAACATGTCGTCCTCCAATGCCTGACCACGGGCCTGGTCAGTCCGGTGATGATCATCCGCAAGGTGGATCGTGCCTCGACCGTGGTCGGCGGCGCCCGAGACGATGTGAGTGGCAGCGGCGGCGAATTTGGCGACGAGGTCTTGGGCGACCCGGTGAGTCAACTGCACAAGATTGCGCTTCAGATCGTGCAAGACCCCAAGATGAGTGTAATGCAAGCCCCCGACCCTCGGATGCCGCGCACCTCTCAGCCGGTGACCTACCTGGCCTGCTTGAATGACATGGTCGGCATGCACAAGACGTCCGAAGGGCGCTCGTGGGCCGGCTGGGACGACAGCATCACCTCGCAGGAAGGAGGCAAGATCATCCGGAAACGTCGGGTGTCGACCGACGTCCAGCCCGAGACCCTGATGTCTTGCATGTCCCTTTCAGATTACCCCCGACGTCGGGTCAACAGCCTCGAGGACCCCGCGCCTTATCTCGCCCGCAAGTCGAGCGTCAGCAGCCTTTCTTCCACCACCAGTCGTCCCCACCTCGGCGCCTTTTGGAGTGAAGACGTCTCGGATGCTGCCGTCTGGACCCTCGTCGGCACCGACTGTGCCACCTACACCTTCTGGTCGCCCTTCCTCGACGATCCCTCCACTCCCCTCTCCACCGGCCCTTTCCCGGCCCTCTCTCACTTTTTTACATCGACCAACAAGTTGGATCACGAACGGTTCCTGACCATGCACGGTGAGAATTTCTCGCGTGATCTCCAGGTCTGGTTTGGCGACGTCAAGGCGAATCACACCGAATACCGAAGTAGGGAATTGATCATCTGTAAGGTGCCTCCGAGACATGAACTGATGGAGGTCAAGAAGGTCTATGGTGATCTTCCGATTCTGTTGGTGAGAGGTGATGGCACGATATGTAAAACCGGTAAATGCTTTTCGTTGTAA

RO3G_07636_new_cDNA(exon 1 extended)
ATGAATTATCCAGAATTATTAACAAATGAAGTTGAATCTATCCCATCCTCTTGGTCTTCTAATTCATCTTTACCACATGTTTCTTTATTCTCTCCAAGTTTTTTAGAAACATTAAAATTAGAAGATGAAAATGATTTTAATACAATACATCCTTCTGTTATCTATCATCATTCACCTCAAACCACCACTATTTCTTCACCCGATCATTCCAATCTATTTAATTTACAAGAAAATAAAATTTATTCAAATCATCAACAAAAGAATCTCATTCAACATTATCTATCCACCAAACAAGGAGAAAAGAAACTGACGATTTTGACGAgtaaagtagctcaaaagagttatggaaatgaaaaaagattcttgtgtcctcctccttctactatcctttctggtacgggtcactggtggacagCAAAACAACATCCACCGAATCTGACAATTCAAATCTCTGGTGAAAAATTAAGTCATCAAGGCACGATCGATTGGTACAAGGATGGCAATCTGTTGGATCAACCTTCTGCCGTTCTTTTAGCAAATCAAGGAAGTAATCTGATTGGTAATTGTGTATCCAAGCAATTGCATGTGAGTGGCGCAGATGAAAAGCGAAGGAAAGCGCAAGTTCAAGTCGAGATTCGATCAGGTCAAGGTACACCGATCGGTATCTTTCATTCAAAACCTATCAAAGTGATCAGTAAACCATCCAAGAAGAGACAGAGTGTTAAAAATATGGATCTCTGTATTCATCATGGCACGACTGTCGCTTTATTCAATCGTGTACGAGCTCAGACCATCTCCACAAAATATCTCGGTGTTTCTTCTCTTGACAGTCAACAAAAGGACAGAGGTACTTGTTTTGTCACTCGAACCACCTCTTGGGATCCATTCCTCATCTACATCGTCGATCTCTCTCGTTCTCCAAACACACCTTCTCCAGTGCCATTCAGTCATCATCCCACCATCGATCACTATCCTCCTCCTCCTGCTATCGCCATTCAAAACCATCAGGGCTCACTTGCCCTTCATTATAATCAACCTGTCGTATTACAGTGTGTCTCTACCGGCCTGGTCAGTCCGGTGTTGATCATCCGTCGAGTAGAGAAAGGTAGCATGGTGATGGGTGGTAATCGTGTGAATGATCTTTCTTATCCTACAGGAGGTGAGTGGGGTGATGAAGCCCTGGGTGATCCTGTCTCTCAACTTCACAAAGTCGGATTTCAAATCGTTCAAGATCCATCCATCGCCCAATACAACAAGTCTACTTTTCAGGAACAGGACAAGTTCTTTTTACCCCCTGTCACCCACTGGACCTTGCCTCAGGCCACTTCTGCTATTAATTATCTCGCCTGTATTCATGATGTCGTCGGAATGCATCGAGTGACGGATGAGAGAAAGATCGTGAGTCGATTTACAACAGAAATCGAAGACATCAAGATGGCTGTTCGTAAACGTCGTCTGTCCTATCAGCAACACAGTACTACAGTCAAAAGCTCTAATCGCCGTCGAGTGAATTCTTTAAATGATGAATTGCTATCAAGACATGTTGGTGGCGACGCTGGTCGTTGTCCTGATCAACCCTTAAATGGTGATTGTTGGACTGAAGATGTTTCGGATTCAGCCGTCTGGACGATTGTCGGTACCAATTCGACTAGCTTTGCCTTCTGGACACCTCCTGATTATTCTAAACCATTCTTTGATCTTCAAGATTTTCCCTATGTTGACTCTATCCAATCATTATCCAGTACAGTCTTGTCTCTCGTAGGTGAACACTTTACATCAGACCTCACCGTTTGGTTTGGTGATGTGCCTTCCATCCAGACGGAATTCAAATCAAGTCAACTTCTCTCCTGTACTGTGCCTGAACGACATGAATTATTGGATAGTTTTGCAACTCAGTTAGACCCCGACACAAGCAGGCATAAAATACCTCTTCTTTTGGTTCAAGAAGATGGCATCATTTATAATTCACTACTGTTTTATTCATTTTAA

Pb_ESTEXT_FGENESHPB_PG.C_10057_new_cDNA(exons 2+3 removed)
ATGAACTCGTCTACACACGTGCTTTCTGAGGAAGTCTTTGGCTCGAGTAAAAAGATTCAATTTGTCCAAACCACATCATCCATGGACTCTCTTCTCAGTGCAGTCGACCGCGCACCTCGCATTAACCTGCCATACACCCAATCTAAGCAGACAATGTCCATCGAAGCTCTTTTAGACACCAATGGACACATCAGCAAATCATCCGCAAAACGCTCCTGGACCTACGACATCACCGACAGATCTCCAAAGACAAAGATTCACCGATCCCAGCTAGAAACCCACTCGCCACCAACAGAGCATTGTCCAGAACCAATCCAGCATTTATCTTTGTCTTCTTCGCTCTTACTCCCCCCACAGCGCCTGTCGACAATCACTTGTCTTCATGCTGCAGTTGCCCAGAAATCGTATGGATCCGAAAAGCGATTCTTGTGCCCTCCACCAGTCGTGACTCTCAAGTCAACAACCCCCGGGCTACGCACAGACTCGCCCATAGTATCGATGTCAGTTGTGTGTGAAAGCATCGACCGACCGGTGGAACAACGCGCCTCTCTTGATGACAATCAATCCGGAAGCTTCAAATATCTCCATGTAACCGGTACCGCAAAGGCTAAACAGTTTTGTCTACGTGTTGGTCTCAGCCACAGCCACAGCCACAGCCACAGCCAATTTCCACCCCTTGGGTCTTCATCTCCCCAACAACAGCAAAACCAACATCATCAGATCAGTCAACCGTTTGCAACATTCTTCTCATCTCCAGTATCAATCATCTCCAAGCCATCTAAAAAGACGGCAAAGGCACGAAATGTATCGACCTGTATCCTGGCCAATTCACCAGTCTCGCTCTTCAATCGAATCAACTCCCAGACTGTACGAACCAAATATATGTCCTCAGACGCCAATCGACTGTGTGCCAAAAGCTCGGCATGGTCAGCATTTAATATCCAGATCATCCGCCAACCAGAAGATGCCGAAGACAACGAGGACGGGCCTGTGCCGGTACTTTACGGAACTGAAATTATTCTAAAAGACACCCAGAGTGGAGTATGTTCTCCACCTCTTATAGTCCGTAAGGTTGACAAGGGCTGTATTGCGGCCACTGCAACAGGGCCCATTAGCCAGATGCAGAAGATTGCACTCCAACTTAAATCCTCTGTTAACAGTCAGCCAACATATCTTAGTGCAGGCGGAAATATCATTACACCAACAGACCAATCTTCAGATCATAACGGCAACACATCATCAGCATCAATAACAACAACATCATTATCATCATCATCATCAGCAGCAGCAGCAGCAGCAGTAGGATCACCATCATCTAATCAAAATTCAAATGCTTGGCTTGATATTTCTCCTTCACAACCATCCAAGAACAACAATGATCCAAAAGTTGAAGTTGTTGATGATTATCTCTGCTGGACGATTGTTTCTATTGCCAAGTTTGAATATACATTTGCAGATCCAATAATGGTGGGAATGGATGTCAGCCCTTGCTCCAAAATGGCTGAATCTCGACTTGTGCCTTCTTCTGCATCGTCTTCCTCGTATTCTTCTCCTTCTCATTCTCCCTCTCCGTCTTCACTGACATGTCGACATGCGCAACCCTCTCCTCCTCCTTCACCACCGCGAACGATTGTACCATACCCTAGTCTGTCTAGTGTTAGCTACAACCACACCTTGCACGCGATTGATGTTGTTGGCCATCATCTGTTCCAAAAGTCAACAATGCCACCTCGGCTGCTTGAATTTTGGTTGGGTAATCATGGTCCCCTCAAGCAGGTTCCTCACGAGACAAGTTTGGTCAGGGTCGAATTGCCACCTACTCAAGATCTCCTTGTGGCCAACCACAATATTCTGCAACGACAAGCGAATGGAGAGCGACATCTTGAATTGGCTCTTGTTCTGGTGCGTCAAGATGGAATGGTCTATCCTACCGGTAAATCTCTATCCTGTGCAGTATCAGTAAATGGAGATACCAGTCGGTGgtatgtcgtgcctacccattcacactccctgtcatga

Pb_FGENESHPB_PG.2__465_new_cDNA(exons 2+3 removed)
ATGCCCAGTATGGCCGATTCTACCTCTTCTCCGTCCTATGGTTGTTCCAGTCAACCACTGTCTCCTGAAATGCAGAATAATCGTAAACGCAAACAGCTACAATACCCCACTTCAGTCGATAATTCGCCTTATATGAACATCCCGTACCCCCCCGCCCAATGGTCAACAGACCCCTATGCTACCCATTCCTCCATCGGATACACCACTGAAAAACCTGATATGATGTCCTGGCCATCAACCCAATTCGACCCTCGTTCTACCTCCACCTCAACTCCAGAAAGCAGAGTTCGTCAACCATCCTCAACATCTTCTGCCACAGTAGACATCGACGACCTTTTTGATCAAATTGACCCAACCCTCATTCCAGGAACATCCTCAAATCCTGCCCGCTCATTCTATGTCTATGATCAAGACCAACCCCCCACAGATCAACAATCGGCCTCAATCATCTCATCGCAACCACAGGCCTGCCAATCGTTTGACTTCATCACTTCATCGTATGCTTATTCTGGACAGTCACTCCAAGACGTCCCACTCGATATCCACTCCTTCCCACTTCGCGAATCCCCTCAGGTGCCCTCCCAGCTCGGCTCAGAACCAAACCAGAACACCAGATACAGGCAATCCGACCTTCAGCTCCACGACCTTCATACTGTCCATCGACGCAACCAGCAACAATTGATTCAGTCCTGGGTTGGTGCAACATACTCAGCTCCCCAACAGGTATCTCCGAATCACTCCCCAGCCGGCTCAAATCCAGCCTTTTCTCCCGTTACTCCCGCCTTCTTCTCTCCCAGTTTCCTCGACTCTCTTCAGGGCGATGGCCATAGCTCACCCAGGCAGCTCACGTTGGACCAGTCGTACTCCCAGCCTGTCTCAACCGGTCTTGACACTCGACCCTTGGACTGGAATTCTGATTATAATTCCACTCAAGCTGATCAAAGACTTAACATGGTTGCCCAGACAAGAGATATGCTCTCACCACCTGCATCACCTCCACACTCGTCAAATTCGTCGACAAATTCGTCTCCTCCAATTACTCCCATGTATGGTCGTTTGTCAAACCTCTCGATTCGCTCCACACCGGTTTCTGTTTCTGTTTCGGCTCCTGGTCAAATCCCATTCAACGGGCATCACTACTACGATAATTCGGCCACTACTACTATCCCAACAAACATGACTGGCGGACATCCGATGCTAGTCGTAGCCACAGCAGCCCATCCACCAACCATTCCAGAAGCGGCGAGTGAAGAAGACGAAACTCGACTAACCTCGGGTCCTATTTCCTCTCCACACCGAAACCACCCAAACGTTATTATTGATCGTCGTTCAGTCAAGACCAATCGACAGATGATTCAAGCCTTTCAAGCCGCAACCTTTCGACCTCACATTCAGAAATATCTCAGGAGCCCAGACCCAATGTCGGTTGGTGAACGTACAGTTGTTATACTCACTTCCAAAGTGGCACAAAAGAGTTATGGTACCGAAAAGAGATTTCTGTGTCCCCCGCCCACTGCCATTCTGGTGGGTACTACTTGGTGGACTCCCAAAAAGGATGCAACGTTAGACGATGGCCATGTCTCGGACGATGCCCAAAGTCAACCACCTGACCCATTTCTCATCGACAATGAACGCGTCCTGGCACCCCCAAGACTGACGGTCTGCATTTCCGGAGAGGCATCAAGCCAGGCTGGACACATCGAATGGTACACTGTCTCGGGTGTGACGGTCGGCCAGACTGGACATGTTAAGGCAGCCCAACCTTCTTCCTCGTCTTCGTCTTCTTCTCCTTCTGGAAACGGCACCACCAGTGGCACCAGTGCTCAACAAGAAGGTCTGCATCCCAATAGATTCAGAAGTGCAGAATCAAGAAACAATAACTTTGATTGGTACCACAACCATCACCAGGAACCCTTGGCTGCCGGAAAGAATGTCTCGAAACATCTGTTTATCAATGATGCCGACGAAAAACGGAAAAGGGTCGAATGTCTGGTCAAGATTCAGTTGGCCAATGGTCTGATGCTTGGGACACTTGCCAGTAAAGGAATCAAGGTCATTAGCAAACCAAGCAAGAAAAGGCAGAGTGTTAAAAACATGGAGCTCTGTATTCATCATGGCACAACTGTATCTCTGTTTAACCGAATCCGCTCTCAGACCGTCTCTACCAAATACCTTGGCGTGTCTTCTTCTTCAGCTCAGGGCTCGCCTTATGCTTTTCCGGGTCATGCTCAACCAAAGACCCAGGCAAGCGATGTCAATGACGGTACCTGTTTTGTAGCCCGAACTACTTGCTGGGATCCCTTTGTGGTCTGGGTGGTGGACACTACGCGCACTCCGGGCGACGGTAAAGAAAGTGACACACCCGAAGATTACATTGGTCGCAATGTGTACACCCCCAGCGTGCCTTACCCTCCGCCACCCGCGATTGCTTTGAAGAACAAGACAAACCAGCTAGTCCCGATTCTCTACAACCAGCACATTGTTCTGCAGTGCCTTACAACCGGTCTTGTTAGCCCGGTTATGATCATACGCAAGGTAGACAAGGCATCTACTGTTGTAGGTGGCGCTCGTTCTGTCGATGACCCTCACATTTCTGGAGGTGGTGAGTATGGCGATGAAGTATTCGGAGACCCTGTTTCCCAACTCCACAAGATTGCCCTTCAGATCGTCCAAGACCCTTCAAATGCAGTACACGCCCATCATGCTGCAATGCGCCAGCAAACCCAATCCCAAAATGGAGACTACGATATGAACTACAGCAATTCTGGTGTATTTTCACCTACGCATCTACCCACACCAGCACCAGCACCAGCACCAGTACCTCAATTGCAAGAAAAAATGATGCCACGCTCCAACCAGCCTGTCACTTACTTGGCATGTCTGAATGATATGGTTGGAATGCACAAGACTGTGGATCCCAGACGACCTTTAGCGACCATATCACAGCAGACAGCAGCAGAGGCAGCCGCGACCGGAGCATACTTTCTGAAAGAGGAGACGATGAATAATTACAATACGATCGATAGTTCGGTTCCTTCGCAGCAAGAAGGTGGGAAAGTAGTCCGTAAGCGACGAGTGTCGACTGATGTTCTTGAAAGACACCTTAATTCCGGTTTGAACAACTACAATTTAAATATGAGTATGAGTATGAATATGAATATGAATATGAACGGAATACCGCCCAGTAAACTCGTTAGTTCAATGTCGCTCACCAACCTCAAGGAAAGAAATCAGGGCGAGATACTTGATCCTAGTCTTCGCCGACGCGTCAATAGTATGAACGATGATATTGAGGCATACTACGGAAGCCGTGCAAATGGCGGAAGTGCAGCATCGCATATTCTCATGAATCGTGGCCGATCAGGGTCGACGCATTCTCTGGAGAACAGTAAGCGAAAGCCAAGCATGAGTGCCAGTAGTAGCTCTAGCTCGACTAGCACCGGCAGCCGTCGTATGTCAGCTGTGAGTGTGAATGCTGGTAGCCCTAACCATGGACTTGGTTCATATTGGAGTGAGGATGTTTCTGACGCAGCAGTTTGGACAATTGTAGGAACAGATTGTGCTGTATATACATTCTGGACACCTCCTGTGGACGACACACGGCCACAGTTGAGCGTAAATACAACAATGTTCCCTACCTTGTTGAACTACACTCTCAATATGTCGCCTAGTGTGGCCTACCAGACGCAGCAAAGCACAGGTCTCGACCGACGCCTTCAATCGACAGGAGAAGAATACCCTGATTCAAATGAACAATTGATGTCCTTGCACGGCGAGTCCTTTTCGCGTGATTTGCAGGTGTGGTTTGGAGACATCAAGGCGCTTGATTCGGATTACCGTAGCCGAGAATTGTTTGTATGCAGGTTACCTACGCGTCAGAACCTCTTGGAGGGAGTAGGACTCGAGACAGTTGGCTTTGACAATACGACACATGCTCCTATCTACAGTCTACCGATTCTATTGGTCCGAGGAGATGGCACTGTTTACAAGACCAACAAGACCTACAAATTCCAGtaa
